# Bioactive Artificial Cells as Autonomous Metabolic Actuators Enable Bidirectional Communication with Tumor Cells

**DOI:** 10.1021/jacs.5c14609

**Published:** 2025-12-12

**Authors:** Lifan Hu, Wenwu Peng, Jiyao Yu, Lisa Förch, Stephen Mann, Seah Ling Kuan, Tanja Weil

**Affiliations:** † 28308 Max Planck Institute for Polymer Research, Ackermannweg 10, Mainz 55128, Germany; ‡ Ulm University, Albert-Einstein-Allee 11, Ulm 89081, Germany; § Centre for Protolife Research and Centre for Organized Matter Chemistry, School of Chemistry, 1980 University of Bristol, Bristol Bs8 1ts, U.K.; ∥ Max Planck Bristol Centre for Minimal Biology, School of Chemistry, Bristol BS8 1TS, U.K.

## Abstract

Artificial cells (ACs) offer a powerful platform to reprogram metabolic signaling in complex tissue environments by replicating key biological functions without the full complexity of living cells. However, achieving autonomous metabolite exchange and stable integration with living tissues remains a major challenge. Here, we report the development of proteinosome-based ACs equipped with a minimal metabolism to mediate bidirectional communication with glycolytic tumor cells. These tumors accumulate lactate, a metabolic byproduct that promotes immunosuppression and metastasis. Although lactate oxidase (LOx) can degrade lactate, its oxidation product, pyruvate, may inadvertently fuel tumor growth. To overcome this limitation, we engineered dual-processor ACs coencapsulating LOx and pyruvate decarboxylase (PDC), enabling selective conversion of lactate into cytotoxic acetaldehyde while suppressing pyruvate and hydrogen peroxide accumulation. These ACs demonstrate sustained catalytic activity, maintain reactive oxygen species homeostasis, and remain functional when integrated in 3D tumor spheroids. Crucially, they engage in autonomous, bidirectional metabolite exchange, preferentially with cancer cells over normal cells, dynamically rewiring important metabolites of the tumor microenvironment and suppressing cell viability. This work establishes synthetic metabolic biointerfaces as programmable actuators capable of reshaping pathological signaling in cancer tissues.

## Graphical abstract

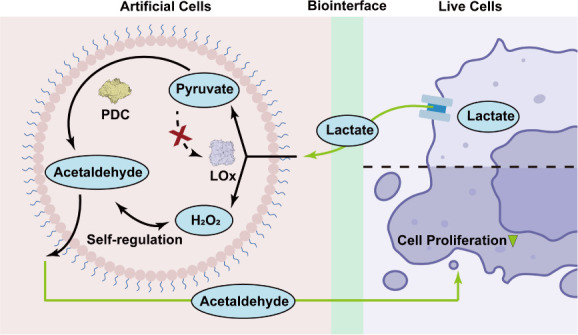

## Introduction

Metabolic reprogramming within the tumor microenvironment (TME) is a hallmark of cancer progression, enabling malignant cells to thrive under stress and evade immune surveillance. In triple-negative breast cancer (TNBC), this reprogramming is especially pronounced,^1^ marked by elevated lactate levels, acidic pH, and oxidative imbalance.^2,3^ These changes not only support tumor growth but also reshape cell–cell communication and reduce the efficacy of therapeutic interventions.

Artificial cells (ACs) have emerged as promising platforms to emulate selected biological functions with high programmability.^4^ By operating as confined, cell-like compartments, ACs can be engineered to carry out defined biochemical reactions, respond to environmental cues, and interact with living systems.^5–10^ Despite recent progress, most AC–cell systems remain limited to one-way signaling,^11–14^ lacking the ability to autonomously sense, respond, and communicate in a dynamic and reciprocal manner.

Lactate is one of the most abundant and functionally relevant metabolites in the glycolytic TME, with concentrations ranging from 10 to 40 mM in tumors,^15,16^ and even exceeding 40 mM in the necrotic core,^17,18^ in contrast to low levels found in normal tissue (1.5–3 mM).^15,19^ The accumulation of lactate promotes angiogenesis, immune suppression, and metastasis.^20–26^ Targeting lactate with enzymatic depletion strategies has therefore gained attention as a means to disrupt tumor-supportive signaling and bolster antitumor immunity.^27,28^ Lactate oxidase (LOx) catalyzes the oxidation of lactate to pyruvate and hydrogen peroxide (H_2_O_2_), offering a direct route to lactate removal.^29^ However, both products introduce new challenges: H_2_O_2_ drives DNA damage and inflammatory signaling,^30–32^ while pyruvate can support tumor cell metabolism,^33–35^ inhibit LOx catalysis,^36,37^ and be recycled back to lactate.^3^ Effective metabolic intervention must therefore not only eliminate lactate but also manage its downstream metabolites. Pyruvate, in particular, remains an underexplored target in metabolic intervention and signal conversion, despite the availability of several enzymes capable of transforming it.^38–40^ For example, pyruvate decarboxylase (PDC), a yeast-derived enzyme, catalyzes the decarboxylation of pyruvate into acetaldehyde (AcH), a known cytotoxic compound.^39^

Here, we develop proteinosome-based ACs equipped with a minimal, dual-enzyme metabolic network that converts tumor-derived lactate into a cytotoxic and redox-buffering outputs within an AC framework for bidirectional communication with cancer cells (Figure 1). By coencapsulating LOx and PDC, these ACs transform lactate into acetaldehyde via pyruvate while simultaneously limiting the accumulation of pyruvate and H_2_O_2_. Acetaldehyde not only suppresses cancer cell proliferation^41–43^ but also reacts with H_2_O_2_ to form α-hydroxy hydroperoxide (α-HHP), thereby buffering oxidative stress.^44,45^ This built-in feedback enables effective lactate clearance while moderating peak ROS levels, creating a selective communication interface that perturbs cancer cell metabolism without inducing uncontrolled oxidative damage to nonmalignant cells. The resulting constructs operate as autonomous metabolic processors that integrate stably into both 2D cancer cell cultures and 3D tumor spheroids. Our findings demonstrate that ACs can serve as synthetic metabolic biointerfaces, enabling bidirectional communication with tumor cells, reprogramming local metabolism, and suppressing malignant behavior in tissue-like contexts. This work represents a conceptual advance in bottom-up synthetic biology, introducing a blueprint for programmable biointerfaces that dynamically interact with and reshape diseased microenvironments.

**Figure 1:**
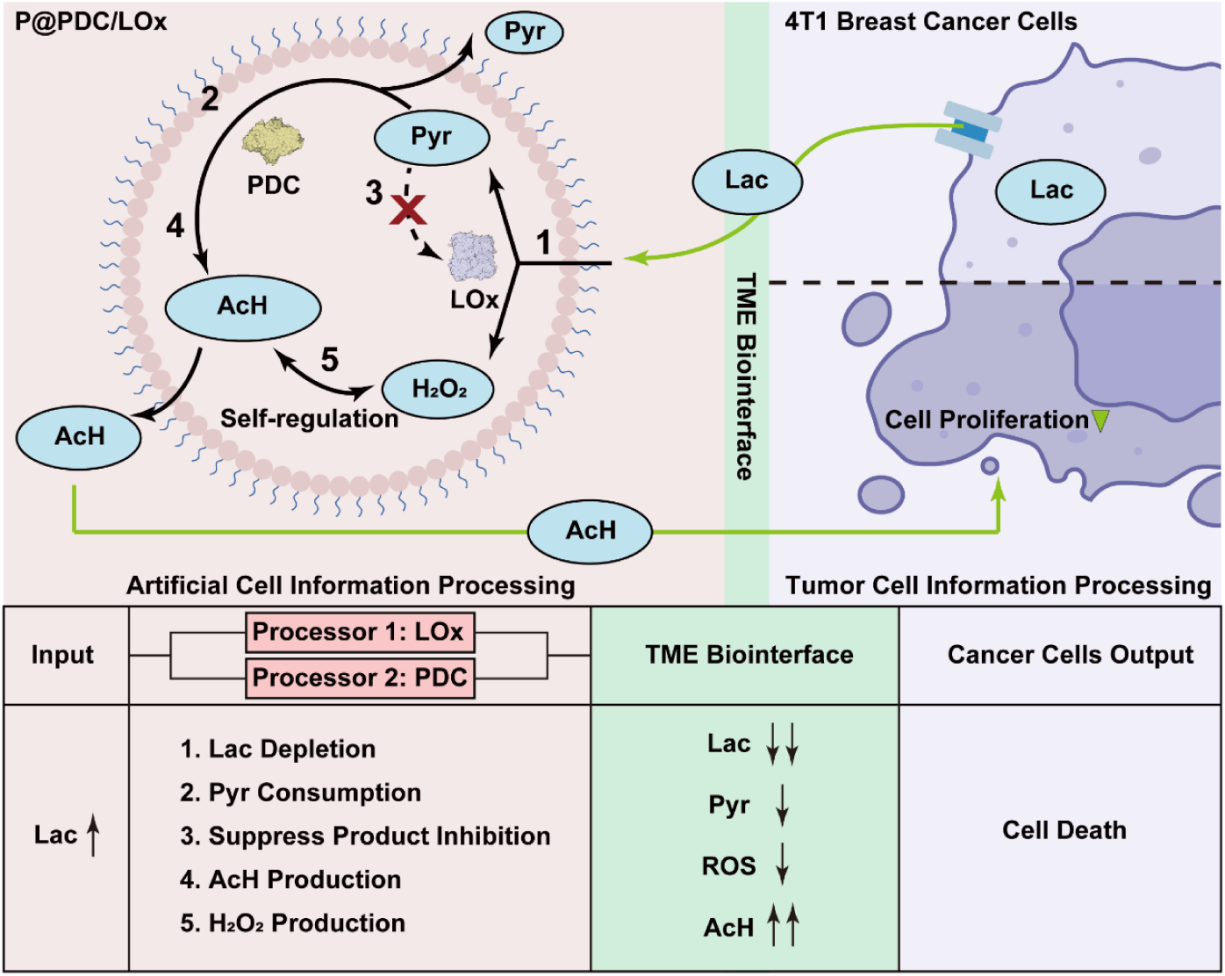
Proteinosome-based artificial cells (ACs) with minimal metabolism convert and produce metabolites relevant to the tumor microenvironment (TME). Bidirectional metabolic crosstalk between P@PDC/LOx and 4T1 breast cancer cells. Top panel: ACs with minimal metabolism process cancer cell-derived lactate via a P@PDC/LOx dual processors (LOx as processor 1 and PDC as processor 2), executing five sequential functions: **1**, LOx-catalyzed lactate to pyruvate and H_2_O_2_ conversion; **2**, PDC-driven pyruvate consumption; **3**, suppression of pyruvate-mediated product inhibition; **4**, PDC-catalyzed AcH production; **5**, H_2_O_2_–AcH homeostatic regulation within ACs decreasing ROS overproduction: without AcH production (P@LOx), H_2_O_2_ levels continuously rise; with AcH production (P@PDC/LOx), H_2_O_2_ levels decrease, supporting homeostatic regulation of ROS. Bottom panel: Metabolic products from P@PDC/LOx (depleted lactate, decreased pyruvate, decreased ROS and elevated AcH) serve as new exogenous input signals for tumor cells. The AC-mediated inputs (i) downregulate lactate production and pyruvate accumulation, thereby disrupting metabolic homeostasis, (ii) suppress cancer cell growth through various AcH-mediated cytostatic and cytotoxic mechanisms; and (iii) modulate oxidative damage by homeostatic regulation of H_2_O_2_ by AcH. Together, these effects trigger cell death. ACs: artificial cells; Lac: lactate; Pyr: pyruvate; AcH: acetaldehyde; P@LOx: LOx encapsulated in ACs; P@PDC/LOx: PDC and LOx coencapsulated within the same ACs; solid arrows: catalyzed reactions or direct molecular interactions; dashed arrows: abolished product inhibition due to substrate depletion. The images of LOx and PDC are created with Biorender.com.

## Results

### Dual Enzyme Processors Convert and Regulate TME-Relevant Metabolites

We established a minimal, dual-enzyme metabolic network responsive to tumor-associated signals, by combining the enzymes LOx and PDC. The cascade functions via sequential signal processing: LOx oxidizes lactate to pyruvate and H_2_O_2_, and PDC decarboxylates pyruvate to AcH (Figure 2A). At mildly acidic pH values representative of the TME, PDC displayed peak activity at pH 6.4, while LOx retained ∼85% activity relative to its maximum at pH 7.4 (Figure S1A,B). AcH, the terminal output of this enzymatic cascade, was maximally produced at pH 6.4 (Figure S1C), aligning with the local pH of proliferating TNBC tissues.^46^ Tuning the PDC:LOx ratio allowed control over the amplitude of AcH product formation, while single-enzyme controls served as comparisons. All assays were performed using lactate concentrations within the linear detection range of the respective assays and, where possible, under pathophysiologically relevant conditions (10–50 mM lactate).^15–18^ With a constant LOx input (5 μg/mL) across experiments, lactate depletion was significantly enhanced in the dual-enzyme (PDC:LOx) groups compared to LOx alone (∼1.4-fold increase; Figure 2B), indicating efficient pyruvate turnover and prevention of pyruvate-induced product inhibition. Pyruvate accumulation was highest in the LOx-only group (272 ± 15 μM), while increasing PDC concentrations led to dose-dependent reductions (down to 205 ± 7 μM with 2 μg/mL PDC), confirming active downstream processing (Figure 2C). PDC alone did not generate pyruvate. Next, we evaluated AcH production at a lactate concentration of 40 mM, which is pathophysiologically relevant and consistent with reported levels in the TME.^15,16^ Notably, AcH, absent in all single-enzyme controls, was exclusively detected in the PDC and LOx conditions (Figure 2E) and upregulated with enzyme stoichiometry, with a 2-fold increase in AcH output for the PDC:LOx 2:1 group relative to 1:1. This demonstrates the ability to program metabolite output through enzyme ratio control.^47^ Beyond signal modulation, the cascade exhibited self-regulating properties: H_2_O_2_ levels, a potentially pro-tumorigenic byproduct of LOx activity,^48^ were markedly suppressed (by 48–85%) in the presence of both enzymes (Figure 2D). This suppression is attributable to the reaction of AcH with H_2_O_2_, as corroborated by ^1^H NMR spectrum of a stoichiometric reaction of acetaldehyde with H_2_O_2_ to form α-hydroxy hydroperoxide (α-HHP)^44^ (Figure S2). Together, these results define a lactate-responsive dual-enzyme system with programmed activation, metabolite amplification, ROS regulation and built-in feedback. AcH is only produced when both enzymes are present (1,1), whereas all other processor combinations (0,0; 0,1; 1,0) fail to generate a productive output (Figure 2F). By dynamically processing and regulating lactate, pyruvate, and H_2_O_2_, this cascade can be applied as a programmable metabolic circuit tailored to the challenges of the TME.

**Figure 2:**
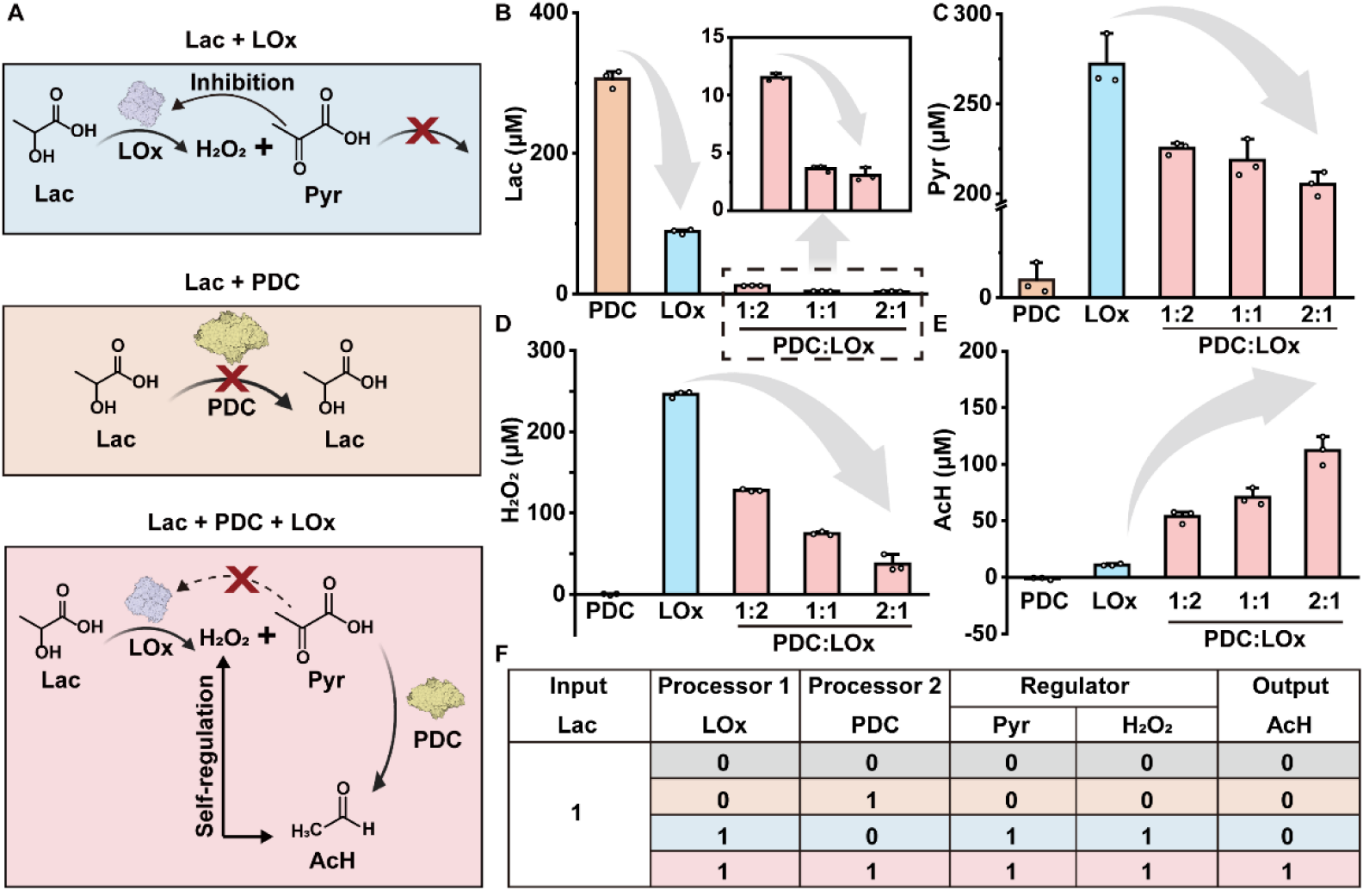
PDC and LOx form a dual processor that converts lactic acid and pyruvate into acetaldehyde (AcH) without H_2_O_2_accumulation. A. Operational sequence of the LOx-PDC cascade: schematic outcomes for single-enzyme (PDC or LOx) and combined (PDC with LOx) treatments in lactate solution. B. Lactate quantification validating LOx-dependent substrate depletion (lactate, 0.3 mM, 1 h incubation at 37 °C in 50 mM phosphate buffer (PB, pH 6.4) with 5 μg/mL LOx and 2.5, 5, or 10 μg/mL PDC, respectively), amplified by PDC-mediated pyruvate removal. Values are plotted as mean ± SEM (*n* = 3). C. Pyruvate levels generated by LOx were proportionally metabolized by PDC (lactate, 0.25 mM; 1 h incubation at 37 °C in 50 mM PB (pH 6.4) with 1 μg/mL LOx and 0.5, 1, or 2 μg/mL PDC, respectively). Values are plotted as mean ± SEM (*n* = 3). D. AcH scavenges H_2_O_2_ through nucleophilic addition, thereby reducing oxidative byproduct accumulation and maintaining ROS homeostasis. Assays were performed with 0.25 mM lactate and incubated for 1 h at 37 °C in 50 mM phosphate buffer (pH 6.4) containing 1 μg/mL LOx and 0.5, 1, or 2 μg/mL PDC, respectively. Aliquots were diluted 5-fold prior to analysis to ensure measurements remained within the validated linear range of the assay. Data are presented as mean ± SEM (*n* = 3). The lactate concentration used in panels B–D was selected to remain within the linear detection ranges of the respective assay kits. E. AcH production occurs exclusively in the presence of both enzymes and scales with the PDC:LOx stoichiometry (lactate, 40 mM; 1 h incubation at 37 °C in 50 mM phosphate buffer (pH 6.4) containing 10 μg/mL LOx and 5, 10, or 20 μg/mL PDC, respectively). Data are presented as mean ± SEM (*n* = 3). The AcH production at this lactate concentration falls within the linear detection range of the assay and reflects pathophysiologically relevant conditions. F. Bienzymatic processing of lactate by LOx and PDC in solution. Table showing enzymatic combinations (LOx, PDC) to achieve output specific formation: In the absence of both enzymes (0,0) or with PDC alone (0,1), no detectable metabolite outputs are observed; with LOx alone (1,0), pyruvate and H_2_O_2_ accumulate but no AcH is produced; with both enzymes present (1,1), pyruvate accumulation is reduced, H_2_O_2_ is maintained at low levels, and AcH is generated. Lac: lactate; Pyr: pyruvate; AcH: acetaldehyde. The images of LOx and PDC are created with Biorender.com.

### Artificial Cells with Minimal Metabolism Sustain Tumor-Relevant Signal Processing

To build ACs capable of processing metabolic signals from the TME, we coencapsulated LOx and PDC into proteinosome (P)-based compartments (P@PDC/LOx) and used lactate as an input signal to initiate the enzymatic conversion to AcH via a two-step endogenous cascade. ACs were fabricated by interfacial assembly of cationic human serum albumin (cHSA)–poly­(*N*-*iso*propylacrylamide) (PNIPAAm) bioconjugates at water–oil interfaces, followed by cross-linking and phase transfer (Figure 3A).^49^ HSA was selected for its physiological compatibility and reduced immunogenicity compared to bovine serum albumin.^50^ Modified cHSA exhibited increased amine content and enhanced grafting density of PNIPAAm (*M*_n_ ≈ 8.2 kDa), enabling robust proteinosome formation (Supplementary Methods, Figures S3–S8). The resulting ACs were narrowly dispersed, spherical (15–30 μm), and structurally stable in aqueous and cell culture environments (Figures 3B, S9–S12). Confocal and scanning electron microscopy confirmed their morphology (Figure S10 and Video S1), while atomic force microscopy (AFM) revealed a membrane thickness of ∼7.5 nm and reversible size changes upon hydration (Figure S11). Importantly, the ACs maintained their structure in oxidative conditions (10–100 μM H_2_O_2_) thatmimick redox stress in the TME,^51,52^ and showed thermoresponsive contraction near the LCST of PNIPAAm (∼32 °C),^53^ with ∼5% size reduction across physiological temperature ranges (Figures S13, S14).

**Figure 3:**
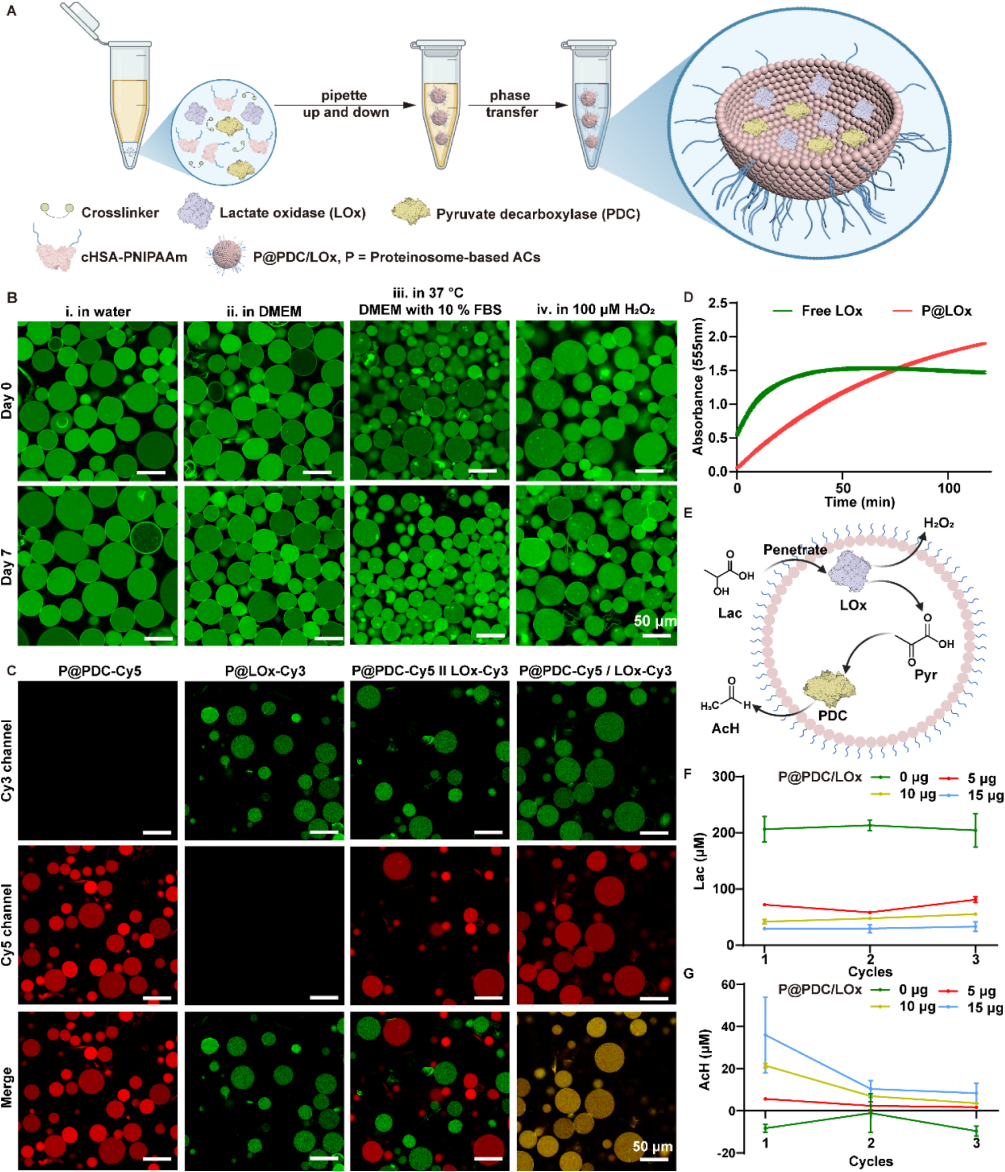
Artificial cells (ACs) containing enzyme processors LOx and PDC (P@PDC/LOx) reveal sustained metabolic activity. A. Schematic of proteinosome-based ACs preparation via interfacial assembly. B. Confocal microscopy images of ACs under different conditions up to 7 days (0.5 μg/μL ACs in IBIDI 18-well plate). i: ACs in water at room temperature; ii: ACs in DMEM with 10% FBS at room temperature; iii: ACs in DMEM with 10% FBS at 37 °C; iv: ACs in 100 μM H_2_O_2_ environment at room temperature. Scale bar = 50 μm. C. Confocal microscopy images of four different AC designs (0.5 μg/μL ACs suspended in Milli-Q). Cy5-labeled PDC (red) and Cy3-labeled LOx (green) exhibit overlapping signals when coencapsulated (P@PDC/LOx ACs (merged)). In contrast, mixed AC populations (P@PDC∥LOx) containing the individual enzymes retain their red and green signals (group P@PDC∥LOx). All groups were imaged after 24 h of incubation. Scale bar = 50 μm. D. Catalytic activity of free LOx and encapsulated LOx (P@LOx), reactions were carried out in 50 mM PB (pH 6.4), using either 0.2 μg/mL free LOx or 17.1 μg/mL of ACs encapsulating 0.3 μg/mL LOx (P@LOx); absorbance was recorded immediately after mixing enzyme and substrate (lactate solution 55.6 mM, 4-aminoantipyrine (4-AA) solution 0.33 mg/mL, *N*-ethyl-*N*-(2-hydroxy-3-sulfopropyl)-*m*-toluidine (EHSPT­(TOOS)) solution 0.26 mg/mL, peroxidase solution 11.1 U/mL). E. Schematic representation of compartmentalized lactate-to-AcH conversion. F. Lactate consumption scales with P@PDC/LOx concentration upon substrate replenishment. Reactions were carried out in 50 mM PB (pH 6.4) containing 0.2 mM lactate, incubated for 1 h at 37 °C, and repeated three times with fresh substrate (0.2 mM lactate in 50 mM PB). 5, 10, 15 μg P@PDC/LOx in 50 μL reaction solution to get 100, 200, 300 μg/mL P@PDC/LOx. Values are plotted as mean ± SEM (*n* = 3). G. AcH production correlates with P@PDC/LOx concentration across three cycles of lactate replenishment. Reactions were carried out in 50 mM PB (pH 6.4) containing 50 mM lactate, incubated for 1 h, and repeated three times with fresh substrate (50 mM lactate in 50 mM PB). 5, 10, 15 μg P@PDC/LOx in 50 μL reaction solution to get 100, 200, 300 μg/mL P@PDC/LOx. The negative values for the control group is due to the detection limit of the assay^67^ (see Supporting Information for calculations). Values are plotted as mean ± SEM (*n* = 3). Lac: lactate; Pyr: pyruvate; AcH: acetaldehyde; P@PDC: PDC encapsulated in ACs; P@LOx: LOx encapsulated in ACs; P@PDC/LOx: PDC and LOx coencapsulated within the same ACs; P@PDC∥LOx: PDC and LOx encapsulated in separated ACs (the mixed populations of P@LOx and P@PDC). Figure 3A and E is created with Biorender.com.

To confirm enzyme encapsulation, we loaded LOx-Cy3 and PDC-Cy5 into ACs and visualized colocalized fluorescence signals via confocal microscopy. Samples were imaged immediately after preparation (Figure S15) and after 12 and 24 h incubation (Figures S16, 3C). Single-enzyme-loaded ACs exhibited isolated fluorescence signals, while coloaded P@PDC/LOx compartments showed overlapping Cy3/Cy5 signals with no evidence of leakage. Mixing separately labeled P@LOx and P@PDC confirmed spatial segregation of enzymes. Within the detection limits of our assay, no enzyme release into the surrounding medium or transfer between adjacent ACs was observed. Encapsulation efficiencies were 21% for LOx and 71% for PDC, as determined by calibration-based fluorescence quantification (Figure S17). Catalytic assays revealed distinct kinetic profiles for free versus encapsulated enzymes (Figure 3D). While free LOx rapidly consumed lactate and plateaued within 35 min, the encapsulated enzyme exhibited sustained linear conversion over 110 min, likely due to substrate diffusion across the proteinosome membrane. Multicycle assays confirmed functional cascade activity: lactate depletion led to transient activity loss, but catalytic turnover was restored upon substrate replenishment (Figure 3F). AcH production remained stable across three cycles (35 μM, 10 μM, and 8 μM), underscoring the durability and scalability of the encapsulated biocatalytic network (Figures 3G, S18). The encapsulated system retained ∼73% of its initial function after 24 h and ∼47% after 7 days, compared with ∼80% and ∼43% retained by the free PDC/LOx mixture at the same time points (Figure S19). Under both conditions, the cascade remained functionally active for up to 1 week.

Together, these results establish P@PDC/LOx as a structurally robust and catalytically active AC capable of repeated metabolite processing in environments mimicking the TME. The dual-enzyme network enables sustained conversion of lactate into downstream products, including cytotoxic AcH, under physiologically relevant and oxidatively stressed conditions.

### Spatial Location Affects Efficiency of Metabolite Signaling in Autocrine- and Paracrine-Like Compartmentalization

To explore how enzyme compartmentalization affects signal propagation and metabolic efficiency, we engineered ACs to emulate autocrine- and paracrine-like signaling modes.^54^ In the autocrine-like configuration (P@PDC/LOx), both enzymes were coencapsulated in the same ACs, enabling self-contained cascade processing. In contrast, the paracrine-like model (P@PDC∥LOx) consisted of spatially segregated ACs, requiring diffusion of the pyruvate intermediate between compartments for full cascade activation (Figure 4A). Lactate consumption was significantly higher in autocrine-like ACs than in paracrine-like systems (final concentrations: 18.0 ± 5.1 μM vs 41.1 ± 9.0 μM; Figure 4B). This enhanced efficiency arises from the short diffusion distance within the same compartment, which minimizes pyruvate loss and overcomes feedback inhibition. In contrast, in the paracrine-like configuration, pyruvate is transferred between the different ACs by diffusion through the external environment, delaying conversion and reducing overall flux.

**Figure 4:**
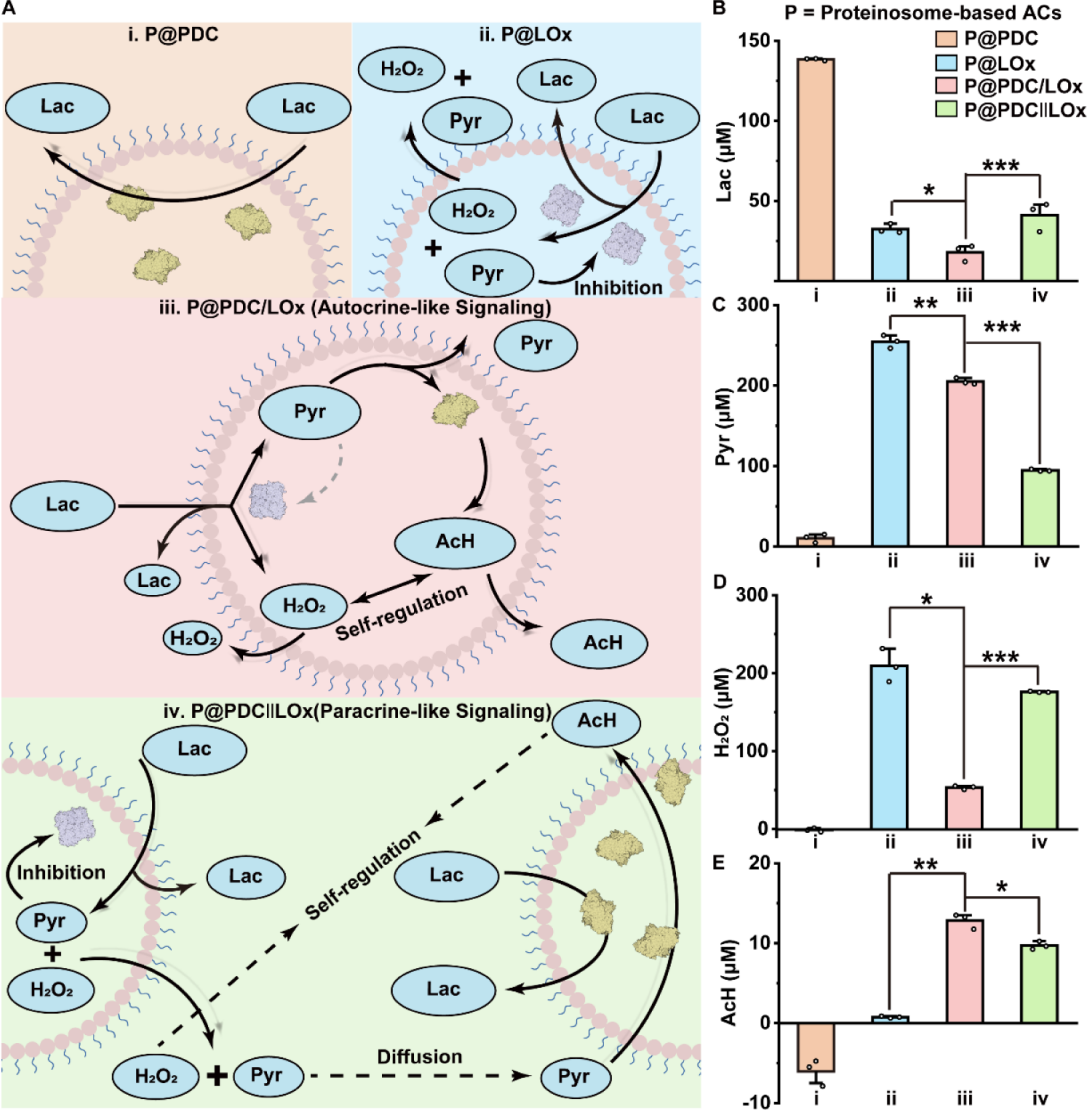
Autocrine- (P@PDC/LOx) versus paracrine- (P@PDC∥LOx) like signaling in engineered AC processors dictate enzymatic reaction cycles. A. Mechanistic pathways for lactate consumption in four different AC models: (i) P@PDC, (ii) P@LOx, (iii) P@PDC/LOx, and (iv) P@PDC∥LOx. Autocrine-like signaling (iii) enhances catalytic efficiency through spatial proximity, enabling rapid intermediate transfer, while paracrine-like signaling (iv) has limited catalytic activity due to diffusion between the compartments. B–E. Changes in lactate (B), pyruvate (C), H_2_O_2_ (D) and AcH (E) concentrations following addition of lactate to the different AC populations. C. Pyruvate concentrations following treatment with four groups. D. H_2_O_2_ concentrations following treatment with four groups. E. AcH concentrations following treatment with four groups. The negative values are due to the detection limit of the assay.^67^ Data in panel (B, C, D, E) were analyzed by a one-way ANOVA with Games–Howell test. N.S.: *p* > 0.05, *p < 0.05, **p < 0.01, and ***p < 0.001. In models (i), (iii), and (iv) the PDC concentration was held constant at 33.6 μg/mL, while in groups (ii), (iii), and (iv) the LOx concentration was 10 μg/mL. Reactions in (B, C, D) were performed in 50 mM PB (pH 6.4) containing 0.18 mM lactate and incubated for 1 h at 37 °C. Reactions in (E) were performed in 50 mM PB (pH 6.4) containing 44 mM lactate, incubated for 2 h at 37 °C. The low lactate concentration was selected in B–D to remain within the linear detection range of the kits. The high lactate concentration was selected in E to evaluate the final output AcH under pathophysiologically relevant conditions. Lac: lactate; Pyr: pyruvate; AcH: acetaldehyde; P@PDC: PDC encapsulated in ACs; P@LOx: LOx encapsulated in ACs; P@PDC/LOx: PDC and LOx coencapsulated within the same ACs; P@PDC∥LOx: PDC and LOx encapsulated in separated ACs (the mixed populations of P@LOx and P@PDC). The images of LOx and PDC are created with Biorender.com.

Pyruvate accumulation further confirmed these spatial effects (Figure 4C). Pyruvate levels were highest in LOx-only ACs (255 ± 8 μM), intermediate in P@PDC/LOx (205 ± 4 μM), and lowest in the paracrine-like P@PDC∥LOx (95 ± 1 μM). The reduced accumulation in the latter suggests feedback-limited LOx activity due to delayed downstream processing. Reactive byproduct analysis revealed further advantages of autocrine-like configuration. H_2_O_2_ levels were significantly lower in P@PDC/LOx (53.2 ± 2.3 μM) compared to paracrine-like ACs (175.8 ± 0.9 μM; Figure 4D), consistent with rapid scavenging via in situ-generated AcH. Correspondingly, AcH production was enhanced in autocrine ACs (12.8 ± 0.9 μM vs 9.7 ± 0.5 μM; Figure 4E), reflecting more complete cascade turnover.

Together, these results demonstrate that ACs with minimal metabolism can be spatially programmed to mimic biological signal architectures. In autocrine-like processors, colocalized enzymes achieve higher cascade fidelity, faster metabolite turnover, and redox self-regulation through feedback-like cross-talk between H_2_O_2_ and AcH. Paracrine-like systems, though less efficient, offer modularity and phase separation, akin to spatially distributed pathways in cellular metabolism and synthetic consortia. This spatial logic control underscores the utility of proteinosomes as customizable artificial cells for dynamic metabolite exchange, redox balancing, and synthetic communication across multicellular environments.

### Artificial Cells Communicate Metabolically with Tumor Cells and Modulate Their Viability

To investigate whether ACs can establish metabolic communication with living tumor cells, we developed a coculture model using enzyme-loaded ACs and 4T1 murine TNBC cells (Figure 5A). In this system, lactate secreted by 4T1 cells acts as an input signal, processed by the encapsulated enzymes within the ACs to generate regulatory metabolites. Nonenzyme-containing ACs were fully biocompatible, maintaining 4T1 cell viability across a range of concentrations (Figure S22A) and showing no effect on extracellular lactate or pyruvate levels (Figure S20). In contrast, enzyme-loaded ACs significantly altered the metabolic landscape. P@PDC/LOx achieved greater lactate depletion than P@LOx (12.4 mM vs 10.0 mM; Figure 5B), reflecting the cascade’s ability to consume pyruvate and relieve product inhibition of LOx. This was supported by pyruvate measurements: P@PDC/LOx generated significantly less pyruvate (175.6 ± 22.6 μM) than P@LOx (506.6 ± 148.5 μM; Figure S21A), enabling sustained catalytic activity. AcH production, absent in all other conditions, was exclusive to P@PDC/LOx cocultures (79.6 ± 5.0 μM; Figure 5C), highlighting the synthetic ACs’ capacity for multistep biocatalysis and bidirectional signaling. An apparent increase in total aldehyde levels was observed in the P@PDC/LOx system during coculture with 4T1 cells (Figure 5C) compared to the cell-free model (Figure 4E). The colorimetric assay (Sigma MAK139) quantifies total aldehydes and is not specific for acetaldehyde.^55^ Notably, 4T1 cells alone exhibited a higher background signal, likely due to aldehydes such as malondialdehyde produced through lipid peroxidation.^56,57^ Thus, the elevated signal in coculture reflects a combination of enzymatically generated acetaldehyde and preexisting cellular aldehydes in the tumor microenvironment. H_2_O_2_ levels in the P@PDC/LOx system (3.7 ± 0.2 μM) were slightly lower than in P@LOx (3.2 ± 0.1 μM; Figure S21B), although this difference did not reach statistical significance (*p* = 0.08). This trend suggests that AcH production may buffer H_2_O_2_ accumulation, consistent with redox regulation observed in solution assays (Figure 4D). Notably, oxidative buffering was less pronounced in cell culture, possibly due to the intrinsic ROS-scavenging capabilities of tumor cells.^58,59^

**Figure 5:**
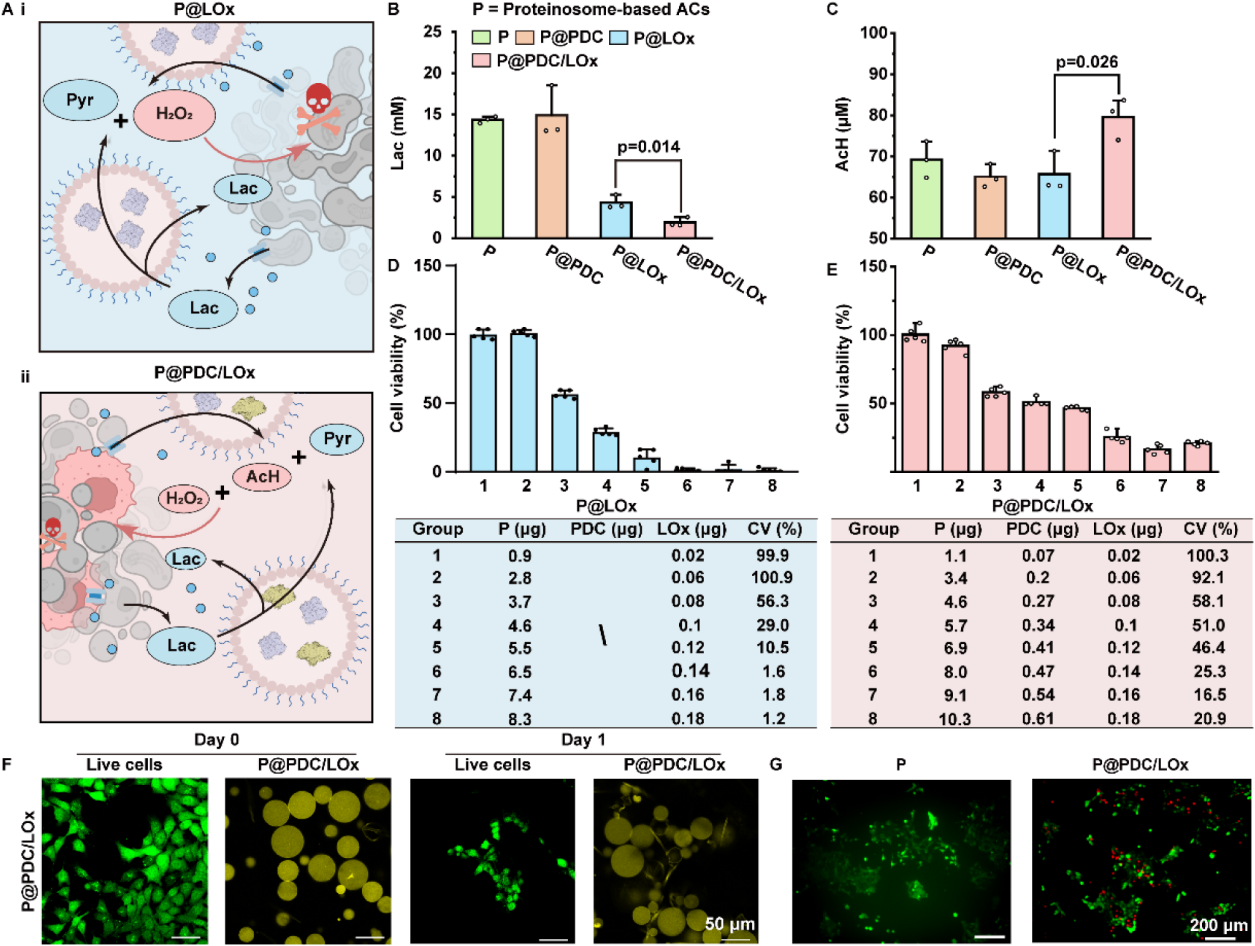
Cocultures of 4T1 cancer cells and artificial cells (ACs) with minimal metabolism reveal a metabolic crosstalk that reduces cancer cell viability. A. Schematic illustration of the dynamic communication circuit established between enzyme-encapsulated ACs and cancer cells. Different AC configurations gave rise to distinct interaction modes. For example, P@LOx ACs supported unidirectional conversion of lactate into pyruvate and H_2_O_2_, acting as oxidative signal generators (i). In contrast, P@PDC/LOx ACs with coencapsulated LOx and PDC enabled a self-regulating cascade that converted lactate into AcH while limiting H_2_O_2_ accumulation (ii), offering a more complex form of metabolite processing. B, C. Lactate (B) and aldehyde (C) concentrations on 4T1 cells level after being treated in cell plates with nonenzymatic ACs (46 μg), P@PDC (44 μg containing 2.7 μg of encapsulated PDC), P@LOx (37 μg containing 0.8 μg LOx), or P@PDC/LOx (46 μg, containing 2.7 μg PDC and 0.8 μg LOx). Values are plotted as mean ± SEM (*n* = 3). These data were analyzed by an independent *t* test. *p* < 0.05 indicates significant difference. D, E. Cytotoxicity of 4T1 cell treated with different concentrations of P@LOx (0.9 μg with 0.02 μg LOx; incubation time, 24 h) and P@PDC/LOx (1.1 μg with 0.07 μg PDC and 0.02 μg LOx; incubation time, 24 h). Values are plotted as mean ± SEM (*n* = 5). F. Confocal images of 4T1 cells incubated with P@PDC/LOx for 0 day and 1 day. Green channel: Cell tracker-green stained cells. Cells plated in 8-well confocal plates were treated with P@PDC/LOx (46 μg ACs containing 2.7 μg PDC and 0.8 μg LOx). Yellow channel: ACs. Scale bar = 50 μm. G. Live/dead staining after nonenzymatic ACs or P@PDC/LOx were incubated with cells after 1 day. Scale bar = 200 μm. Cells plated in 96-well plates were treated with 11.5 μg of nonenzymatic ACs and P@PDC/LOx (11.5 μg ACs containing 0.68 μg PDC and 0.2 μg LOx). Lac: lactate; Pyr: pyruvate; AcH: acetaldehyde; P@PDC: PDC encapsulated in ACs; P@LOx: LOx encapsulated in ACs; P@PDC/LOx: PDC and LOx coencapsulated within the same ACs; CV: cell viability. Figure 5A is created with Biorender.com.

Cytotoxicity assays revealed that both LOx-loaded ACs reduced 4T1 viability in a dose-dependent manner, whereas empty ACs were nontoxic (Figure 5D,E). P@LOx exerted stronger cytotoxic effects, consistent with unregulated H_2_O_2_ release. P@PDC/LOx showed lower cytotoxicity, reflecting its internal feedback mechanism that tempers ROS levels through AcH-mediated scavenging. Confocal microscopy showed that ACs maintained structural integrity and were nondisruptive during early interactions (10 min), with 4T1 cells exhibiting normal morphology (Figures 5F, S23, S24). After 24 h, P@LOx and P@PDC/LOx triggered cytoplasmic retraction and altered morphology, indicative of stress responses. Live/dead staining (Figures 5G and S25) revealed that P@LOx induced widespread membrane rupture, while P@PDC/LOx produced a mixed population of live and dead cells, consistent with moderated ROS signaling. This is consistent with live-cell imaging where we observed that empty ACs supported continuous 4T1 proliferation (Video S2), whereas P@PDC/LOx induced progressive cell death (Video S3).

To assess the specificity of the metabolic intervention, we performed parallel coculture experiments using mouse fibroblast L929 cells as a model of nontumorigenic, healthy cells. In contrast to highly glycolytic 4T1 cells, L929 cells produced substantially lower baseline extracellular lactate (1.2 mM, Figure S26 vs 14.3 mM, Figure 5B). Their morphology remained unchanged across all treatment groups, including P@LOx and P@PDC/LOx (Figure S27), and their viability after 24 h coincubation with either formulation remained high (≳90% by CellTiter assay, Figure S28). This likely reflects the lactate-scarce microenvironment of L929 cells, which limits both lactate consumption by ACs and downstream aldehyde formation. Compared with the pronounced cytotoxicity observed in 4T1 cells under identical conditions (Figure 5D–G), these findings demonstrate that enzyme-loaded ACs preferentially perturb tumor cells that maintain a high-lactate microenvironment through two-way metabolic communication. Importantly, control experiments with L929 fibroblasts showed only minor lactate changes, minimal downstream metabolite formation, and preserved viability, confirming that this lactate-responsive cascade selectively targets glycolytic tumor cells while sparing healthy cells. Together, these findings demonstrate that enzyme-loaded ACs can engage in metabolic crosstalk with tumor cells, shaping the TME and influencing cellular viability. The P@PDC/LOx configuration functions as a bidirectional processor, converting endogenous, tumor-derived lactate into cytotoxic and regulatory output, while maintaining redox balance. This programmable interaction mirrors key features of cell–cell communication and provides a blueprint for developing synthetic metabolic interfaces for therapeutic applications, where cancer and healthy cells coexist.

### Artificial Cells Suppress Tumor Spheroid Growth via Autonomous Metabolic Crosstalk

To explore the capacity of enzyme-loaded ACs with autocrine metabolism (P@PDC/LOx) to modulate tumor metabolism under physiologically relevant conditions, we established cocultures with 4T1 breast cancer spheroids. These 3D models better mimic *in vivo* tumor biology than 2D monolayers, capturing key features such as metabolic gradients, extracellular matrix (ECM) interactions, and spatial cell organization.^60,61^ We tested two strategies to integrate ACs into the spheroid environment: (i) direct mixing and (ii) core–shell encapsulation,^62^ with or without Matrigel as an ECM-mimetic scaffold to enhance AC retention and structural stability in the 3D cocultures. Without ECM support, direct mixing led to fragmented spheroids and peripheral accumulation of ACs postwashing (Figures 6C, S29A), indicating poor AC retention in the tumor spheroids. In contrast, *in situ* embedding of the ACs within the Matrigel improved structural stability and facilitated uniform AC distribution around intact spheroids in both strategies (Figure 6A–C). However, direct mixing of the enzyme-loaded ACs (P@PDC/LOx) with Matrigel disrupted spheroid formation, while the core–shell approach produced reliable cocultures without compromising spheroid architecture (Figure 6D). Consequently, core–shell assembly in the presence of Matrigel was selected for further studies. Moreover, this platform supported metabolic exchange between ACs and tumor cells over extended periods. Live–dead staining revealed distinct cytotoxic responses across conditions (Figure 6E). Control spheroids (untreated group) or cocultured with nonenzymatic ACs, exhibited classical viability gradients, with live cells at the oxygen-rich periphery and necrotic zones at the hypoxic core (Figure 6E).^63,64^ In contrast, spheroids treated with P@PDC/LOx ACs showed diffuse cell death across the structure and marked volume reduction (∼48% relative to the control group; Figure S30), consistent with growth inhibition triggered by metabolic disruption (Figure 6).^65,66^

**Figure 6:**
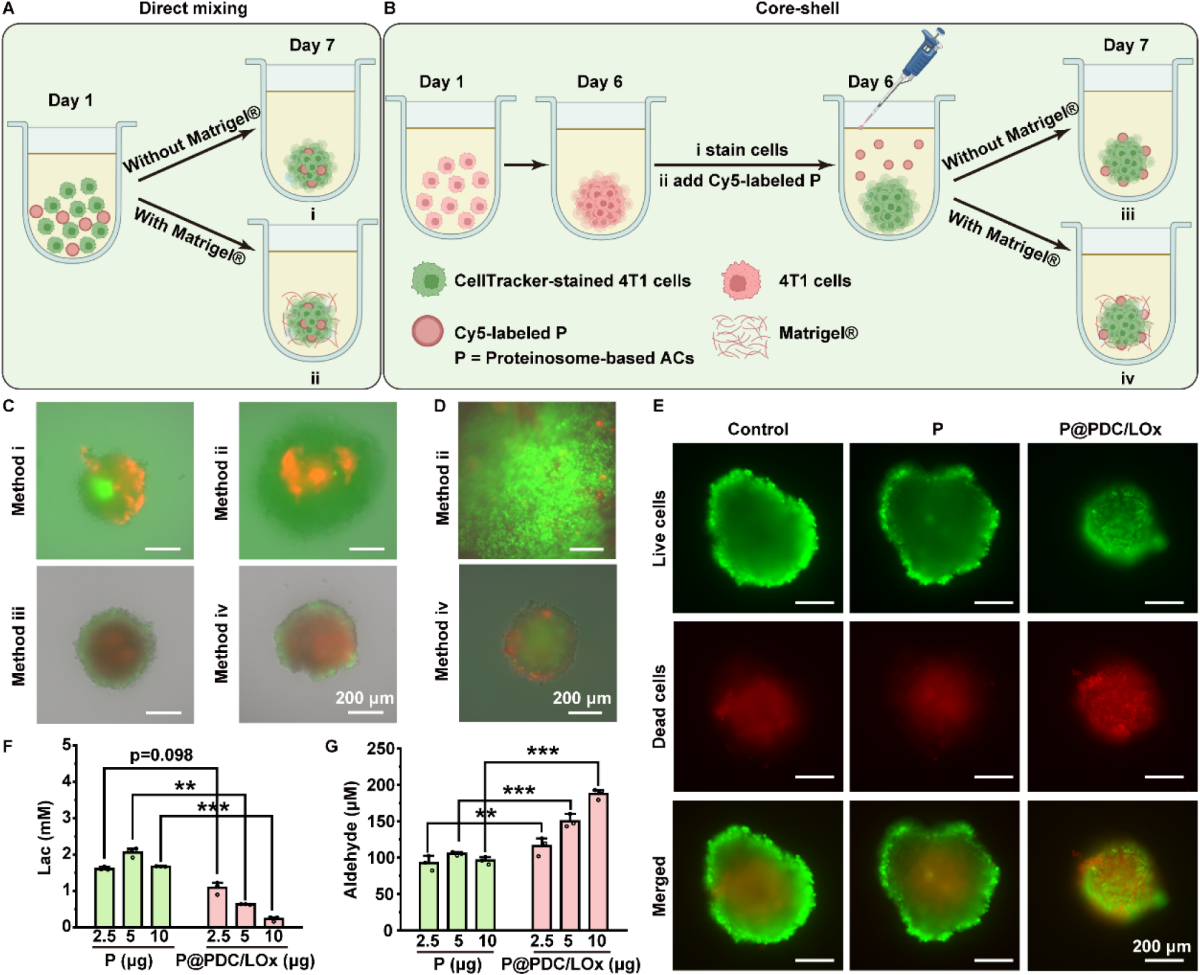
Coculture of 4T1 spheroids with ACs (P@PDC/LOx) reveals a dynamic, autonomous bidirectional metabolite exchange that reduces spheroid viability and growth. A. Schematic of direct mixing coculture methods: (i) without Matrigel and (ii) with Matrigel reinforcement. B. Schematic of core–shell coculture strategies: (iii) without Matrigel and (iv) with Matrigel reinforcement. C. Comparative analysis of postwash structural integrity of 3D cocultures using nonenzymatic ACs and methods (i), (ii), (iii) and (iv): Scale bar = 200 μm. D. Enzyme-encapsulated ACs (P@PDC/LOx) compatibility assessment: core–shell architectures (method iv) maintained structural integrity during enzymatic activity while fragmented spheroids were obtained from direct mixing (method ii). Scale bar = 200 μm. E. Live/dead staining (green: viable, red: dead) reveals spatial cytotoxicity divergence: untreated and nonenzymatic ACs spheroids maintain hypoxic core-restricted necrosis, whereas P@PDC/LOx-treated spheroids exhibit transection-wide cell death. Scale bar = 200 μm. F, G. Metabolic profiling demonstrating P@PDC/LOx-mediated lactate depletion (F) and aldehyde accumulation (G). Values are plotted as mean ± SEM (*n* = 3). Data in panel (F) was analyzed by a one-way ANOVA with Games–Howell test. Data in panel (G) was analyzed by a one-way ANOVA with LSD test. N.S.: *p* > 0.05, *p < 0.05, **p < 0.01, and ***p < 0.001. Core–shell with Matrigel (method iv) was used in E–G; spheroids were assembled from a population of 10,000 cells and treated with 46 μg of nonenzymatic ACs or enzyme-loaded ACs contains 2.7 μg of PDC and 0.8 μg of LOx; incubation time was 24 h at 37 °C, 5% CO_2_. Figure 6A is created with Biorender.com.

Quantitative metabolite analysis confirmed that P@PDC/LOx-treated spheroids exhibited substantial lactate depletion (down to 0.23 ± 0.06 mM with 10 μg P@PDC/LOx; Figure 6F) and AcH accumulation (187.6 ± 7.3 μM with 10 μg P@PDC/LOx; Figure 6G), indicating active biocatalysis and successful cascade operation within the 3D TME. These outputs establish a feedback loop in which tumor-derived lactate fuels enzymatic processing, yielding cytotoxic metabolites that reshape the TME and impair spheroid viability.

Collectively, these findings demonstrate that synthetic ACs with minimal metabolism can stably integrate into tumor-like environments and engage in autonomous, bidirectional metabolic communication. This AC-tumor spheroid hybrid architecture enables precise, sustained reprogramming of local tumor metabolism and introduces a new paradigm for constructing AC–tissue interfaces with therapeutic potential.

## Conclusion

This work introduces a new class of ACs that engage in autonomous, bidirectional metabolite communication with cancer cells, reprogramming the TME through selective metabolic feedback. By integrating a dual-enzyme cascade within proteinosome-based ACs, we demonstrate sustained enzymatic activity, dynamic response to endogenous tumor signals, and feedback-regulated cytotoxicity. Unlike conventional therapeutic carriers, these ACs are not passive delivery vehicles but active participants in metabolic exchange. Their ability to sense, convert, and relay biochemical signals establishes a blueprint for synthetic metabolic interfaces that functionally couple with living cells. The capacity to operate in 3D spheroids further supports their translational relevance and robustness in tissue-like conditions. Our findings define a conceptual advance in bottom-up synthetic biology, positioning ACs as programmable metabolic actuators with potential in precision oncology and regenerative medicine. Future designs could incorporate multi-input sensing, spatial control, and adaptive logic functions, extending the scope of artificial cell technologies toward dynamic control of disease-associated microenvironments.

## Supplementary Material









## Notes

Detailed experimental materials and procedures and supplementary figures (PDF)
Confocal and scanning electron microscopy of resulting ACs (Video S1)
Empty ACs supported continuous 4T1 proliferation (Video S2)
P@PDC/LOx induced progressive cell death (Video S3)
