# Supplementary material for: Bioactive Artificial Cells as Autonomous Metabolic Actuators Enable Bidirectional Communication with Tumor Cells
Source: J Am Chem Soc. 2025 Dec 12;147(51):47255–65. doi: 10.1021/jacs.5c14609 (PMC12752458; doi:10.1021/jacs.5c14609)
Supplement: Supplementary file 1 [file ja5c14609_si_001.pdf]

Bioactive Artificial Cells as Autonomous Metabolic Actuators Enable Bidirectional Communication with Tumor Cells

Lifan Hu [a], Wenwu Peng [a, b], Jiyao Yu [a], Lisa Förch [a], Stephen Mann [c,d], Seah Ling Kuan [a*], Tanja Weil [a, b*]

[a] Max Planck Institute for Polymer Research, Ackermannweg 10, 55128 Mainz, Germany
[b] Ulm University, Albert-Einstein-Allee 11, 89081 Ulm, Germany
[c] Centre for Protolife Research and Centre for Organized Matter Chemistry, School of Chemistry, University of Bristol, Bristol BS8 1TS, UK
[d] Max Planck Bristol Centre for Minimal Biology, School of Chemistry, Bristol BS8 1TS, UK

*Corresponding Author Email: weil@mpip-mainz.mpg.de; kuan@mpip-mainz.mpg.de

Supporting Information

Contents

1. Experimental materials and procedures 3

1.1 Materials 3

1.2 Equipment 3

1.3 Experiments 4

1.3.1 Lactate concentrations tested by lactate assay kit (MAK064) 4

1.3.2 Pyruvate concentrations tested by pyruvate assay kit (MAK332) 6

1.3.3 H_2O_2 concentrations tested by hydrogen peroxide assay kit (MAK166) 7

1.3.4 Acetaldehyde concentrations tested by colorimetric aldehyde assay kit (MAK139) 8

1.3.5 Enzyme activity test 10

1.3.6 Synthesis of mercaptothiazoline activated 4,4'-azobis (4-cyanovaleric acid) (ACVA-ACPM) 13

1.3.7 Synthesis of bis(propylsulfanylthiocarbonyl) disulfide 14

1.3.8 Synthesis of RAFT-agent 14

1.3.9 Synthesis of PNIPAAm....15

1.3.10 Synthesis of cationic HSA (cHSA)....15

1.3.11 Synthesis of cHSA-PNIPAAm and BODIPY (BDP)-cHSA-PNIPAAm....16

1.3.12 SDS-PAGE analysis....16

1.3.13 Proteinosomes-based artificial cells (ACs) preparation....17

1.3.14 SEM, AFM samples preparation....17

1.3.15 Stability of proteinosomes-based ACs in different environment....18

1.3.16 Confocal of LOx-Cy3 and PDC-Cy5 encapsulation....18

1.3.17 Storage stability of free PDC/LOx and PDC/LOx encapsulated in proteinosome-based ACs....19

1.3.18 Cytotoxicity of proteinosomes-based ACs on 4T1 and L929 cells....20

1.3.19 Co-culture with 4T1 and L929 2D cells....20

1.3.20 Live/dead staining....20

1.3.21 Development of spheroid–proteinosome co-culture systems....21

1.3.22 Live dead staining of spheroid–proteinosome co-culture systems....21

1.3.23 Statistical analysis....22

2. Supporting figures....23

3. Reference....45

1. Experimental materials and procedures

1.1 Materials

Human serum albumin (HSA), hydrogen chloride (HCl), 1-Ethyl-3-(3-dimethylaminopropyl) carbodiimide hydrochloride (EDC·HCl), dimethyl sulfoxide (DMSO), tris(2-carboxyethyl) phosphine hydrochloride (TCEP), sodium acetate, dithiothreitol (DTT), lactate, pyruvate, Pyruvate Decarboxylase from baker′s yeast (S. cerevisiae), 4-aminoantipyrine (4-AA), N-ethyl-N-(2-hydroxy-3-sulfopropyl)-m-toluidine (EHSPT(TOOS)), β-nicotinamide adenine dinucleotide (β-NADH), peroxidase from horseradish, O,O′-Bis[2-(N-Succinimidyl-succinylamino, 2,2′-Azobis(2-methylpropionitrile) (AIBN), carbon disulfide, iodine, 2-ethyl-1-hexanol, Alcohol Dehydrogenase from Saccharomyces cerevisiae, 1,4-dioxane, N-isopropylacrylamide (NIPAM), N,N'-Dicyclohexylcarbodiimide (DCC), 4-Dimethylaminopyridine (DMAP), sodium hydride (NaH, 60 % dispersion in mineral oil), lactate assay kit (MAK064), pyruvate assay kit (MAK332), fluorimetric hydrogen peroxide assay kit (MAK166), colorimetric aldehyde assay kit (MAK139) were purchased from Sigma Aldrich. BDP® FL maleimide, Cyanine5 NHS ester (Cy5-NHS) and Cyanine3 NHS ester (Cy3-NHS) were purchased from Lumiprobe. Lactate oxidase (LCO-301) was purchased from Toyobo Co., Ltd. Matrigel Matrix was purchased from Corning Life Sciences. Ethylendiamine was purchased from Acros Organics. 4,4′-azobis (4-cyanovaleric acid) (ACVA), 2-Thiazoline-2-thiol, 1-Propanethiol, Propidium Iodide (PI) and Fluorescein Diacetate (FDA) were purchased from Thermo Fisher Scientific. Coomassie blue staining was purchased from Bio-Rad. CellTiter-Glo® Luminescent Cell Viability Assay was purchased from Promega. Cell line was obtained from American Type Culture Collection (ATCC, USA). Cell culture media (Dulbecco's modified Eagle's medium (DMEM), and fetal calf serum were from Gibco Life Technologies (Karlsruhe, Germany).

1.2 Equipment

NMR spectra were recorded on Bruker Avance 400 MHz NMR spectrometer. MALDI-ToF spectra were acquired on a Bruker Time-of-flight MS rapifleX MALDI-ToF-MS equipped with a 10 kHz

scanning smartbeam 3D laser (Nd:YAG at 355 nm) and a 10-bit 5 GHz digitizer. Confocal Laser Scanning Microscopy was performed on a STELLARIS 8 Leica DMi8 microscope (Leica Microsystems). The fluorescence images were acquired using a Keyence BZX800 microscope (Keyence, Japan) equipped with the appropriate excitation and emission filters. The SDS-PAGE analysis was conducted by Mini-PROTEAN Precast Gel (Bio-Rad Laboratories, Hercules, CA, USA) and a ChemiDoc Imaging system (Bio-Rad Laboratories, Hercules, CA, USA). Absorbance, luminescence, and fluorescence measurements were conducted using a Tecan Spark 20M multimode microplate reader (Tecan Trading AG, Switzerland) or NanoDrop 2000/2000c spectrophotometer (Thermo Fisher Scientific, Wilmington, Delaware, USA)

1.3 Experiments

1.3.1 Lactate concentrations tested by lactate assay kit (MAK064)

Dilute 1 μL of the 100 nmol/μL lactate standard with 99 μL of lactate assay buffer to generate a 1 nmol/μL standard solution. Add 0, 1, 2, 3, 4, 5 and 6 μL of the 1 nmol/μL lactate standard to prepare 20 μL final solution containing 0 (blank), 1, 2, 3, 4, 5 and 6 μL nmol/well standards. **Free enzymes in lactate solution:** For each reaction group, a 100 μL solution was prepared in 50 mM phosphate buffer (pH 6.4) contained 0.3 mM lactate. The following groups were established: (i) 0.5 μg LOx, (ii) 0.5 μg PDC, (iii) 0.5 μg LOx and 0.25 μg PDC, (iv) 0.5 μg LOx and 0.5 μg PDC, and (v) 0.5 μg LOx and 1 μg PDC. The mixtures were incubated at 37 °C with shaking (200 rpm) for 1 h. **Proteinosomes-based ACs (P) encapsulated enzymes in lactate solution:** Prepare 5, 10, 15 μg P@PDC/LOX in 50 μL phosphate buffer contained 0.25 mM lactate (50mM, pH 6.4). The mixtures were incubated at 37 °C with shaking (200 rpm) for 2.5 h. **Three cycles of lactate consumption:** For the first cycle, prepare 5, 10, 15 μg P@PDC/LOX in 60 μL phosphate buffer contained 0.25 mM lactate (50mM, pH 6.4), and then add additional PB to make the final volume is 75 μL. The final lactate concentration of 0.25 × (60/75) = 0.2 mM. Incubate the tube at 37 °C for one hour. After one hour, centrifuge the tube at 6,000 xg for 5 mins, and then take 60 μL of supernatant as the sample for the first cycle. For second and third cycle, add fresh 60 μL phosphate buffer contained

0.25 mM lactate (50mM, pH 6.4), and repeat the procedures. **Enzymatic cascade influenced by spatial organization:** Add 20.36 μL of P@PDC (contain 54.5 μg ACs with 3.36 μg PDC) into 70 μL phosphate buffer (50mM, pH 6.4) contained 0.25 mM lactate, and then add 9.64 μL PB to get 100 μL sample solution. Add 6.16 μL of P/LOx (contain 46.2 μg ACs with 1 μg LOx) into 70 μL phosphate buffer (50mM, pH 6.4) contained 0.25 mM lactate, and then add 23.84 μL PB to get 100 μL sample solution. Add 17.04 μL of P@PDC/LOX (contain 56.8 μg ACs with 3.36 μg PDC and 1 μg LOx) into 70 μL phosphate buffer (50mM, pH 6.4) contained 0.25 mM lactate, and then add 12.96 μL PB to get 100 μL sample solution. For the group P/LOx with P@PDC, mixing 20.36 μL of P@PDC (contain 54.5 μg ACs with 3.36 μg PDC) and 6.16 μL of P@LOx (contain 46.2 μg ACs with 1 μg LOx) into 70 μL phosphate buffer (50mM, pH 6.4) contained 0.25 mM lactate, and then add 3.48 μL PB to get 100 μL sample solution. The final lactate concentration of 0.25 × (70/100) = 0.18 mM. The sample solutions were incubated at 37 °C with shaking (200 rpm) for 1 h. **Lactate level of 4T1 and L929 cells:** 4T1 cells (50,000 cells/well) or L929 cells (50,000 cells/well) were seeded in 24-well plate for overnight and then treated with varying groups (empty ACs, P@PDC, P@LOx and P@PDC/LOX) for 24 hours. The treatments were 1. empty ACs 46 μg, 2. P@PDC (44 μg ACs containing 2.7 μg PDC), 3. P@LOx (37 μg ACs containing 0.8 μg LOx), 4. P@PDC/LOX (46 μg ACs containing 2.7 μg PDC and 0.8 μg LOx). After 24 hours, collecting the cells medium in the tube for next analysis. Centrifuge the tube at 3,000 rpm for 5 min to remove the cells. For 4T1 cells: Take the supernatant in the tube, and then dilute the supernatant by 100 times with PBS. For 4T1 cells: Take the supernatant in the tube, and then dilute the supernatant by 25 times with PBS. **Lactate level of spheroids:** The core–shell method was employed with Matrigel supplementation: 10,000 cells per well were seeded and centrifuged at 1200 rpm for 5 min, and the spheroids were cultured for six days. On the subsequent day, the complete medium was replaced with 100 μL of medium containing 2.5, 5, 10 μg of empty ACs or enzyme-loaded ACs 2.5, 5, 10 μg, and the plate was centrifuged at 1200 rpm for 5 min. Then, complete medium containing 5% Matrigel (final concentration 2.5%) was then added to each well, followed by centrifugation at 1000 rpm for 5 min at 4 °C. After that, the plate was put into incubator again for 24 hours incubation. After 24 hours culturing, the medium in the wells was took for and dilute by 100 times with PBS for further analysis. Prepare master reaction mix as working solution, containing 23 parts of lactate assay buffer, 1 part

of lactate enzyme mix and 1 part of lactate probe. Take 20 µL of every group, including lactate standards and samples to a 384 well plate. Add 20 µL of working solution into every well. Mix well using a horizontal shaker or by pipetting, and incubate the reaction for 30 minutes at room temperature. Protect the plate from light during the incubation. Measure the absorbance at 570 nm (A570) through Tecan.

1.3.2 Pyruvate concentrations tested by pyruvate assay kit (MAK332)

Prepare a 500 µM standard premix by mixing 10 µL of the 25 mM standard and 490 µL of ultrapure water. Dilute 500 µM standard solution with ultrapure water to get 0, 50, 100, 150, 200, 300, 400, 500 µM standard solution for 20 µL. **Free enzymes in lactate solution:** For each reaction group, a 100 µL solution was prepared in 50 mM phosphate buffer (pH 6.4) contained 0.25 mM lactate. The following groups were established: (i) 0.1 µg LOx, (ii) 0.1 µg PDC, (iii) 0.1 µg LOx and 0.05 µg PDC, (iv) 0.1 µg LOx and 0.1 µg PDC, and (v) 0.1 µg LOx and 0.2 µg PDC. The mixtures were incubated at 37 °C with shaking (200 rpm) for 1 h. **Proteinosomes-based ACs (P) encapsulated enzymes in lactate solution:** Prepare 5, 10, 15 µg P@PDC/LOX in 50 uL 0.25 mM lactate (50mM, pH 6.4). The mixtures were incubated at 37 °C with shaking (200 rpm) for 2.5 h. **Enzymatic cascade influenced by spatial organization:** Add 20.36 µL of P@PDC (contain 54.5 µg ACs with 3.36 µg PDC) into 70 µL phosphate buffer (50mM, pH 6.4) contained 0.25 mM lactate, and then add 9.64 µL PB to get 100 µL sample solution. Add 6.16 µL of P@LOx (contain 46.2 µg ACs with 1 µg LOx) into 70 µL phosphate buffer (50mM, pH 6.4) contained 0.25 mM lactate, and then add 23.84 µL PB to get 100 µL sample solution. Add 17.04 µL of P@PDC/LOX (contain 56.8 µg ACs with 3.36 µg PDC and 1 µg LOx) into 70 µL phosphate buffer (50mM, pH 6.4) contained 0.25 mM lactate, and then add 12.96 µL PB to get 100 µL sample solution. For the group P@LOx with P@PDC, mixing 20.36 µL of P@PDC (contain 54.5 µg ACs with 3.36 µg PDC) and 6.16 µL of P@LOx (contain 46.2 µg ACs with 1 µg LOx) into 70 µL phosphate buffer (50mM, pH 6.4) contained 0.25 mM lactate, and then add 3.48 µL PB to get 100 µL sample solution. The final lactate concentration of $0.25 \times (70/100) = 0.18$ mM. The sample solutions were incubated at 37 °C with shaking (200 rpm) for 1 h. **Pyruvate level of cells:** 4T1 cells were seeded in 24-well plate for overnight and then treated with varying groups (empty ACs, P@PDC, P@LOx and P@PDC/LOX)

for 24 hours. The treatments were 1. empty ACs 46 μg, 2. P@PDC (44 μg ACs containing 2.7 μg PDC), 3. P@LOx (37 μg ACs containing 0.8 μg LOx), 4. P@PDC/LOX (46 μg ACs containing 2.7 μg PDC and 0.8 μg LOx). After 24 hours, collecting the cells medium in the tube for next analysis. Centrifuge the tube at 3,000 rpm for 5 min to remove the cells. The supernatant was then diluted by 15 times with PBS. For preparing the reaction mix as working solution by mixing enzyme mix and dye reagent (94:1) in a clean tube. Take 3 μL of every group, including pyruvate standards and samples to a 384 well plate. Add 27 μL of working solution into every well. Mix well using a horizontal shaker or by pipetting, and incubate the reaction for 30 minutes at room temperature. Protect the plate from light during the incubation. Measure the absorbance at 570 nm (A570) with Tecan.

1.3.3 H_2O_2 concentrations tested by hydrogen peroxide assay kit (MAK166)

Add 22.7 μL of 3% H_2O_2 solution to 977 μL of assay buffer to prepare a 20 mM H_2O_2 stock solution. Add 1 μL of the 20 mM stock solution to 399 μL of assay buffer to get a 50 μM H_2O_2 standard solution. Further dilute the 50 μM H_2O_2 working solution to prepare 50, 10, 3, 1, 0.3, 0.1, 0.03 and 0 μM standards. **Free enzymes in lactate solution:** For each reaction group, a 100 μL solution was prepared in 50 mM phosphate buffer (pH 6.4) contained 0.25 mM lactate. The following groups were established: (i) 0.1 μg LOx, (ii) 0.1 μg PDC, (iii) 0.1 μg LOx and 0.05 μg PDC, (iv) 0.1 μg LOx and 0.1 μg PDC, and (v) 0.1 μg LOx and 0.2 μg PDC. The mixtures were incubated at 37 °C with shaking (200 rpm) for 1 h. **Proteinosomes-based ACs (P) encapsulated enzymes in lactate solution:** Prepare 5, 10, 15 μg P@PDC/LOX in 50 uL 0.25 mM lactate (50mM, pH 6.4). The mixtures were incubated at 37 °C with shaking (200 rpm) for 2 h. **Enzymatic cascade influenced by spatial organization:** Add 20.36 μL of P@PDC (contain 54.5 μg ACs with 3.36 μg PDC) into 70 μL phosphate buffer (50mM, pH 6.4) contained 0.25 mM lactate, and then add 9.64 μL PB to get 100 μL sample solution. Add 6.16 μL of P@LOx (contain 46.2 μg ACs with 1 μg LOx) into 70 μL phosphate buffer (50mM, pH 6.4) contained 0.25 mM lactate, and then add 23.84 μL PB to get 100 μL sample solution. Add 17.04 μL of P@PDC/LOX (contain 56.8 μg ACs with 3.36 μg PDC and 1 μg LOx) into 70 μL phosphate buffer (50mM, pH 6.4) contained 0.25 mM lactate, and then

add 12.96 μL PB to get 100 μL sample solution. For the group P@LOx with P@PDC, mixing 20.36 μL of P@PDC (contain 54.5 μg ACs with 3.36 μg PDC) and 6.16 μL of P@LOx (contain 46.2 μg ACs with 1 μg LOx) into 70 μL phosphate buffer (50mM, pH 6.4) contained 0.25 mM lactate, and then add 3.48 μL PB to get 100 μL sample solution. The final lactate concentration of 0.25 × (70/100) = 0.18 mM. The sample solutions were incubated at 37 °C with shaking (200 rpm) for 2 hours. **H_2O_2 concentrations of cells:** 4T1 cells were seeded in 24-well plate for overnight and then treated with varying groups (empty ACs, P@PDC, P@LOx and P@PDC/LOX) for 24 hours. The treatments were 1. empty ACs 46 μg, 2. P@PDC (44 μg ACs containing 2.7 μg PDC), 3. P@LOx (37 μg ACs containing 0.8 μg LOx), 4. P@PDC/LOX (46 μg ACs containing 2.7 μg PDC and 0.8 μg LOx). After 24 hours, collecting the cells medium in the tube for next analysis. Take the supernatant in the tubes for further testing. For preparing the working solution by mixing Infra-red Peroxidase Substrate Stock 5 μL, 20 units/mL Peroxidase Stock 20 μL, Assay Buffer 475 μL. Take 15 μL of every group, including H_2O_2 standards and samples to a 384 well plate. Add 15 μL of working solution into every well. Mix well using a horizontal shaker or by pipetting, and incubate the reaction for 5 minutes at room temperature. Measure the fluorescence intensity at (λ_{ex} = 640/ λ_{em} = 680 nm) using a fluorescence plate reader (Tecan).

1.3.4 Acetaldehyde concentrations tested by colorimetric aldehyde assay kit (MAK139)

Dilute 10 μL of the 10 mM standard with 90 μL of assay buffer to prepare a 1,000 μM standard solution. Further dilute the 1,000 μM standard solution with dilution buffer to generate 0 (blank), 1, 3.3, 10, 33, 100, 333, and 1,000 μM standards. **Free enzymes in lactate solution:** For each reaction group, a 100 μL solution was prepared in 50 mM phosphate buffer (pH 6.4) contained 40 mM lactate. The following groups were established: (i) 1 μg LOx, (ii) 1 μg PDC, (iii) 1 μg LOx and 0.5 μg PDC, (iv) 1 μg LOx and 1 μg PDC, and (v) 1 μg LOx and 2 μg PDC. The mixtures were incubated at 37 °C with shaking (200 rpm) for 1 h. **Proteinosomes-based ACs (P) encapsulated enzymes in lactate solution:** Prepare 5, 10, 15 μg P@PDC/LOX in 50 uL 62.5 mM lactate (50mM, pH 6.4). The mixtures were incubated at 37 °C with shaking (200 rpm) for 2h. **Enzymatic cascade influenced by spatial organization:** Add 20.36 μL of P@PDC (contain 54.5 μg ACs with 3.36 μg PDC) into

70 μL phosphate buffer (50mM, pH 6.4) contained 62.5 mM lactate, and then add 9.64 μL PB to get 100 μL sample solution. Add 6.16 μL of P@LOx (contain 46.2 μg ACs with 1 μg LOx) into 70 μL phosphate buffer (50mM, pH 6.4) contained 62.5 mM lactate, and then add 23.84 μL PB to get 100 μL sample solution. Add 17.04 μL of P@PDC/LOX (contain 56.8 μg ACs with 3.36 μg PDC and 1 μg LOx) into 70 μL phosphate buffer (50mM, pH 6.4) contained 62.5 mM lactate, and then add 12.96 μL PB to get 100 μL sample solution. For the group P@LOx with P@PDC, mixing 20.36 μL of P@PDC (contain 54.5 μg ACs with 3.36 μg PDC) and 6.16 μL of P@LOx (contain 46.2 μg ACs with 1 μg LOx) into 70 μL phosphate buffer (50mM, pH 6.4) contained 62.5 mM lactate, and then add 3.48 μL PB to get 100 μL sample solution. The final lactate concentration of 62.5 × (70/100) = 44 mM. The sample solutions were incubated at 37 °C with shaking (200 rpm) for 2 hours. **Three cycles of acetaldehyde production:** For the first cycle, prepare 5, 10, 15 μg P@PDC/LOX in 60 μL phosphate buffer contained 62.5 mM lactate (50mM, pH 6.4), and then add additional PB to make the final volume is 75 μL. Incubate the tube at 37 ℃ for 1 hour. After one hour, centrifuge the tube at 6,000 xg for 5 mins, and then take 60 μL of supernatant as the sample for the first cycle. For second and third cycle, add fresh 60 μL phosphate buffer contained 62.5 mM lactate (50mM, pH 6.4), and repeat the procedures. The final lactate concentration of 62.5 × (60/75) = 50 mM. The standard curve was defined by the equation y = 0.001x + 0.0557. The absorbance of control groups fell near the y-intercept (0.0557). Therefore, when the measured absorbance was below this baseline, the calculated acetaldehyde production yielded a slightly negative value, as detailed in Table S1. **Acetaldehyde level of cells:** 4T1 cells were seeded in 24-well plate for overnight and then treated with varying groups (empty ACs, P@PDC, P@LOx and P@PDC/LOX) for 24 hours. The treatments were 1. empty ACs 46 μg, 2. P@PDC (44 μg ACs containing 2.7 μg PDC), 3. P@LOx (37 μg ACs containing 0.8 μg LOx), 4. P@PDC/LOX (46 μg ACs containing 2.7 μg PDC and 0.8 μg LOx). After 24 hours, collecting the cells medium in the tube for next analysis. Centrifuge the tube at 3,000 rpm for 5 min to remove the cells. Take the supernatant in the tube for further experiments. **Acetaldehyde level of spheroids:** The core–shell method was employed with Matrigel supplementation: 10,000 cells per well were seeded and centrifuged at 1200 rpm for 5 min, and the spheroids were cultured for six days. On the subsequent day, the complete medium was replaced with 100 μL of medium containing 2.5, 5, 10 μg of empty ACs or enzyme-loaded ACs 2.5,

5, 10 μg, and the plate was centrifuged at 1200 rpm for 5 min. Then, complete medium containing 5% Matrigel (final concentration 2.5%) was then added to each well, followed by centrifugation at 1000 rpm for 5 min at 4 °C. After that, the plate was put into incubator again for 24 hours incubation. After 24 hours culturing, the medium in the wells was took for further analysis. For preparing the e master reaction mix as working solution by adding 500 μL of assay solution to 2 mg of aldehyde detection reagent. Take 20 μL of every group, including acetaldehyde standards and samples to a 384 well plate. Add 20 μL of working solution into every well. Mix well using a horizontal shaker or by pipetting, and incubate the reaction for 30 minutes at room temperature. Measure the absorbance at 405 nm (A405) through Tecan.

Table S1. A. Concentration–response curve for acetaldehyde measured at 405 nm (Aldehyde Assay Kit, MAK139). B. Calculation of control values. Values for each group were calculated using the formula: (Absorbance - 0.0557) / 0.001. Absorbance < 0.0557 produces slightly negative values.

A

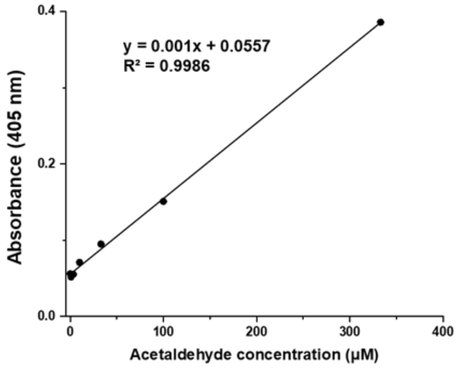

B

	0 μg P@PDC/LOx	Absorbance	Value calculated by the standard line (μM)
the 1st cycle	sample 1	0.0525	-3.2
	sample 2	0.0506	-5.1
	sample 3	0.0515	-4.2
the 2nd cycle	sample 1	0.0601	4.4
	sample 2	0.0543	-1.4
	sample 3	0.051	-4.7
the 3rd cycle	sample 1	0.0522	-3.5
	sample 2	0.05	-5.7
	sample 3	0.0504	-5.3

1.3.5 Enzyme activity test

LOx activity testing (U/mg): To determine the specific activity of commercially purchased LOx (U/mg) used in the work, we used the TOYOBO colorimetric protocol. Prepare four different kinds of working solution (A. lactate solution 0.125 M, B. 4-AA solution 0.5 %, C. EHSPT(TOOS)

solution 5.91 mg/mL, D. peroxidase solution 25U/mL). Mix with 2 mL of solution A, 0.3 mL of solution B, 0.2 mL of solution C and 2 mL of ultrapure water as working solution. Enzyme samples were diluted such that 5 μL contained 0.15 μg LOx; 5 μL of sample was added to 100 μL working solution, mixed, and 30 μL of the reaction mixture transferred to wells of a 384-well plate (n=3). Measure the absorbance at 555 nm (A555) through Tecan to record the increased changes. By calculation, the LOx sample tested had a specific activity of approximately 64.1 U/mg. **LOx activity testing in different pH environment:** Prepare four different kinds of working solution (A. lactate solution 0.125 M, B. 4-AA solution 0.5 %, C. EHSPT(TOOS) solution 5.91 mg/mL, D. peroxidase solution 25U/mL) in 50 mM PB. Mix with 2 mL of solution A, 0.3 mL of solution B, 0.2 mL of solution C and 2 mL of ultrapure water as final test solution. For enzymes activity in different pH test, dissolve the different groups with 50 mM PB of different pH (5.4, 6.4, 7.4, 8.4). Add 500 uL of final test solution into 25 uL sample solution (containing 0.2 μg LOx). For testing the catalysis activity of free LOx and P@LOx: The working solution preparation is the same as above. For the free LOx group, adding 0.1 μg LOx into 25 μL 50 mM pH 6.4 PB; and for P@LOx group, add 9 μg of ACs encapsulating 0.16 μg LOx into 25 μL 50 mM pH 6.4 PB. Measure the absorbance at 555 nm (A555) through Tecan to record the increased changes. **PDC activity testing (U/mg):** The activity of commercially purchased PDC used in the work was measured using a coupled ADH/NADH assay adapted from the supplier's protocol (Sigma P9474). Prepare three different kinds of working solution (A. pyruvate 1 M, B. 6.4 mM β-NADH, C. 200U/mL ADH). Mix with 270 μL of buffer solution, 10 μL of solution A, 5 μL of solution B and 5 μL of solution C to prepare final test solution (290 μL working solution). Mix the 290 μL working solution with 10 μL enzyme solution (containing 2.5 μg PDC). Taking 30 μL of the reaction mixture transferred to wells of a 384-well plate (n=3). Measure the absorbance at 340 nm (A340) through Tecan to record the increased changes. By calculation, the PDC sample tested had a specific activity of approximately 3.6 U/mg. **PDC activity testing in different pH environment:** Prepare three different kinds of working solution (A. pyruvate1 M, B. 6.4 mM β-NADH, C. 200U/mL ADH). For enzymes activity in different pH test, prepare reaction buffer environment with 50 mM PB of different pH (5.4, 6.4, 7.4, 8.4), and add 1 μg PDC in every condition. Mix with 270 μL of buffer solution, 10 μL of solution A, 5 μL of solution B and 5 μL of solution C to prepare final test solution. Measure the

absorbance at 340 nm (A340) through Tecan to record the decreased changes. **K_M and k_{cat} of LOx in P@PDC/LOx:** Prepare a 500 mM lactate stock by dissolving 225 mg lactate in pH 7.5 buffer, then prepare the substrate series 300, 200, 100, 10, 3, 1, 0.3, 0.1 and 0 mM. Prepare 4-AA 0.5% (5 mg/mL), EHSPT(TOOS) 20 mM (5.91 mg/mL), and a peroxidase stock (25 U/mL) and dilute to the working peroxidase concentration; then make the working solution by mixing 500 μL lactate solution (appropriate concentration for each assay well) + 75 μL 4-AA + 50 μL EHSPT + 125 μL peroxidase solution + 500 μL H_2O to give the working reagent. Add 30 μL working solution per well and add enzyme so each well contains 10 μg ACs (in 5 μL, containing 0.175 μg LOx). Immediately start kinetics by measuring A_{555} every 30 s and record the initial linear rate from the early time points. Fit the rate versus lactate concentration to the Michaelis–Menten equation through the software Origin to obtain K_m (mM) and V_{max}. The catalytic constant (k_{cat}) was derived from V_{max} according to the equation $k_{cat} = V_{max} / [E]$. For this calculation, V_{max} was converted to units of $mol·L^{-1}·s^{-1}$ and divided by [E], the molar concentration of active LOx. Perform three independent assays and present the mean ± S.E.M values in Table S2. **K_M and k_{cat} of PDC in P@PDC/LOx:** Prepare a 300 mM pyruvate stock in pH 6.4 buffer and use it to make the substrate series (300, 100, 30, 10, 5, 3, 1, 0.3, and 0 mM). Pipette 30 μL of the appropriate pyruvate solution into each well and add enzyme so that each well receives 30 μg ACs (in 5 μL; containing 1.775 μg PDC). In parallel, prepare acetaldehyde standards (0, 10, 25, 50, 150, 250, 500, 1000 μM) and prepare the acetaldehyde working reagent as described in Section 1.3.4. Add the working reagent to assay wells, immediately begin absorbance testing at 405 nm, and record absorbance every 20 s (or at an appropriate interval) to capture the initial linear phase. Use the acetaldehyde standard curve to convert initial absorbance slopes to acetaldehyde production rates, then fit initial rate versus pyruvate concentration to the Michaelis–Menten equation in Origin to obtain K_m (mM) and V_{max}. The k_{cat} was derived from V_{max} according to the equation $k_{cat} = V_{max} / [E]$. For this calculation, V_{max} was converted to units of $mol·L^{-1}·s^{-1}$ and divided by [E], the molar concentration of active PDC. Perform three independent assays and present the mean ± S.E.M values in Table S3.

Table S2. Summary of enzyme suppliers, catalog numbers, manufacturer-reported specific activities, and experimentally determined activities. Conditions: 37 °C; pH 7.5 for LOx and pH 6.0

for PDC; detection wavelength: 555 nm (LOx) and 340 nm (PDC); reaction volume: 30 μL in a 384-well plate. Mean ± S.E.M. (n = 3).

	Lactate Oxidase (LOx)	Pyruvate Decarboxylase (PDC)
Supplier	TOYOBO	Sigma-Aldrich
Catalog Number	LCO-301	P9474
Reported Activity	80.0 U/mg	5.0–20.0 U/mg
Tested Activity	64.1 ± 0.5 U/mg	3.6 ± 0.8 U/mg

Table S3. Kinetic parameters for LOx and PDC encapsulated in ACs. Michaelis–Menten parameters (K_M, V_{max}) and k_{cat} were obtained from non-linear fits to initial-rate data collected from three independent replicates. Values are plotted as mean ± S.E.M (n = 3).

Enzyme	K_m	V_{max}	k_{cat}
LOx	1.9 ± 0.4 mM	8.0 ± 0.2 mM/min	4247.1 ± 126.4 s^{-1}
PDC	54.2 ± 20.2 mM	4.7 ± 1.0 μM/s	22.0 ± 4.8 s^{-1}

1.3.6 Synthesis of mercaptothiazoline activated 4,4'-azobis (4-cyanovaleric acid) (ACVA-ACPM)

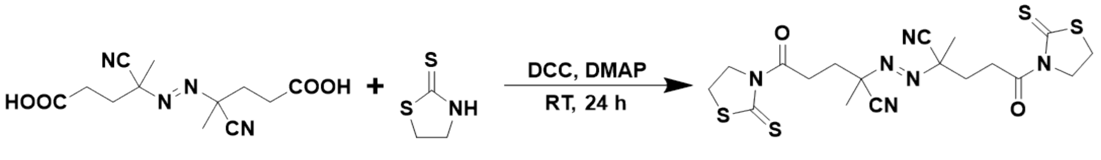

The synthesis procedures from 1.3.6 to 1.3.9 were carried out with reference from the literature [1, 2]. 4,4′-azobis (4-cyanovaleric acid) (ACVA, 2 g, 7.14 mmol, Mw=280 g/mol) and 2-thiazoline-2-thiol (2.3 g, 19.3 mmol, Mw=119 g/mol) were dissolved in 80 mL of 1,4-dioxane, put them together in a 250 mL round-bottom flask. The solution was degassed under a nitrogen (N_2) atmosphere for 30 min. In a separate flask, 1,4-dioxane to dissolve DCC (3.4 g, 16.5 mmol, Mw=206 g/mol) and DMAP (0.05 g, 0.41 mmol, Mw=122 g/mol) were dissolved in 40 mL of 1,4-dioxane. This solution was then added dropwise to the reaction mixture under continuous stirring. The reaction was carried out at room temperature for 24 h. After completion, the reaction mixture was filtered, and the filtrate was concentrated under reduced pressure. The crude product was precipitated in cold diethyl ether and washed with cold diethyl ether three times. The final product was dried under vacuum overnight,

yielding ACVA-ACPM as a yellow powder (2.50 g, 73% yield). ^1H NMR (400 MHz, $CDCl_3$) δ 4.66 – 4.50 (m, 4H), 3.50 – 3.17 (m, 8H), 2.65 – 2.47 (m, 4H), 1.78 (s, 6H).

1.3.7 Synthesis of bis(propylsulfanylthiocarbonyl) disulfide

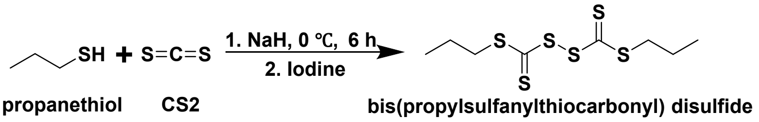

Propanethiol (1.45 g, 19.1 mmol, Mw = 76 g/mol) was added dropwise to a stirred suspension of sodium hydride (NaH, 60% dispersion in mineral oil, 0.79 g, 19.8 mmol, Mw = 24 g/mol) in diethyl ether (40 mL) at 0 °C. The mixture was stirred for 10 min before the addition of carbon disulfide (CS_2, 1.50 g, 19.8 mmol, Mw = 76 g/mol), followed by stirring for an additional 30 min. The reaction mixture was then centrifuged, and the supernatant was collected. The solvent was evaporated under reduced pressure, and the residue was redissolved in 25 mL of diethyl ether. Iodine was added to the solution, and the reaction was allowed to proceed for 1 h under continuous stirring. The reaction mixture was then centrifuged, and the resulting precipitate was washed three times with diethyl ether until it became white. The supernatant was collected and extracted with an aqueous sodium thiosulfate solution to remove residual iodine, followed by drying over anhydrous sodium sulfate. The solvent was evaporated under reduced pressure, yielding the final product as a red oil (81% yield). ^1H NMR (400 MHz, $CDCl_3$) δ 3.32 – 3.27 (m, 4H), 1.74 (h, J = 7.4 Hz, 4H), 1.02 (t, J = 7.4 Hz, 6H).

1.3.8 Synthesis of RAFT-agent

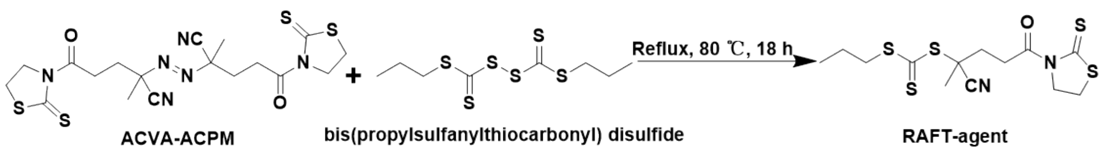

Bis(propylsulfanylthiocarbonyl) disulfide (1.0 g, 3.3 mmol, Mw= 302 g/mol) and ACVA-ACPM (2.4g, 4.9 mmol, Mw = 482 g/mol) were weighed into a round-bottom flask. The system was maintained under an N_2 atmosphere to protect the reaction. Separately, ethyl acetate (40 mL) was degassed under N_2 for 15 min. The solvent was then added to the reaction flask via a syringe, and

the mixture was heated to reflux at 80 °C under an N_2 atmosphere for 18 h. Upon completion, the solvent was removed, and the crude product was purified by flash chromatography using n-hexane/ethyl acetate (3:1) as the mobile phase to afford the product as an orange-red oil (49% yield). ^{1}H NMR (400 MHz, $CDCl_3$) δ 4.58 (t, J = 7.5 Hz, 2H), 3.66 – 3.49 (m, 2H), 3.32 (td, J = 7.3, 1.1 Hz, 4H), 2.55 (dddd, J = 53.1, 14.4, 10.1, 5.6 Hz, 2H), 1.89 (s, 3H), 1.74 (h, J = 7.3 Hz, 2H), 1.02 (t, J = 7.3 Hz, 3H).

1.3.9 Synthesis of PNIPAAm

Weigh RAFT-agent (18.2 mg, 8.2 μmol, Mw = 378 g/mol), N-isopropylacrylamide (NIPAM) (0.905 g, 8.01 mmol, Mw = 113 g/mol) and 2,2′-Azobis(2-methylpropionitrile) (AIBN) (3.28 mg, 0.02 mmol, Mw = 164 g/mol) were weighed into a Schlenk tube. Subsequently, 3 mL of 1,4-dioxane was added. The system was degassed via three freeze-pump-thaw cycles. The reaction mixture was then heated to 70 °C and stirred for 8 h under an inert atmosphere. For purification, a hexane/diethyl ether (1:2) solution was pre-cooled at −20 °C. The polymerization solution was added dropwise into the pre-cooled mixture, and the resulting precipitate was collected by centrifugation. The precipitate was washed three times with the same hexane/diethyl ether solution. Residual solvent was removed by drying under an air pump overnight, yielding a light-yellow powder with a 92% yield.

1.3.10 Synthesis of cationic HSA (cHSA)

The processes of cHSA synthesis followed as described before [1]. In brief, 10 mg of HSA dissolved in 1 mL of 2.5 M degassed ethylenediamine solution and the pH was adjusted to 4.75 through HCl. Secondly, adding 51.07 mg of EDC·HCl into protein solution and the final solution was stirred for two hours at room temperature. Finally, acetate buffer (4 M, pH 4.75) was utilized to stop the reaction. The reaction solution was purified once with acetate buffer (100 mM, pH 4.75) and three times with deionized water in a 30 kDa amicon-ultra tube. The resulting solution was frozen dry to get solid product, the molecular weight is 72,800 Da from MALDI.

1.3.11 Synthesis of cHSA-PNIPAAm and BODIPY (BDP)-cHSA-PNIPAAm

cHSA-PNIPAAm: 2 mL of PNIPAAm solution (2 mg/mL, Mw = 8215 g/mol) was dissolved in aqueous carbonate buffer (10 mM, pH 8.0) and added dropwise to 2 mL of cHSA (2 mg/mL) in the same aqueous carbonate buffer. The mixture was stirred at room temperature overnight. The resulting solution was purified using a 30 kDa amicon ultra centrifugal filter against Milli-Q water at 4 °C. The purified bioconjugates were obtained by lyophilization.

BDP-cHSA-PNIPAAm: 5 mg of cHSA (Mw 85,122) was dissolved in 2 mL of urea-PB (50mM PB, 2 mM EDTA, 5M urea) and stirred for 15 min. After 15 min, 8.4 µL 10 µg/µL TCEP (Mw 286), was added into solution to denature cHSA for additional 30 min. After denaturing, BDP® FL maleimide (Mw 414, 6.1 µL, 20 µg/µL in DMSO) was added and stirred overnight at room temperature. Finally, the reaction mixture was purified by ultrafiltration with urea-PB (10 mM phosphate buffer, pH 7.4, 5 M urea and 2 mM EDTA) three times and with deionized water for several times until no yellow color in the filtrate, then lyophilized to obtain BDP-cHSA-PNIPAAm as yellow solid. Through testing the absorbance by Nanodrop, the information about BDP® FL maleimide and the formula: 1. Protein concentration = (A_{280}-(A_{max} × CF)) × dilution factor / ε; 2. Moles dye per mole protein = A_{max} of the labeled protein × dilution factor / ε' × protein concentration. About 1.2 moles dye modified on per mole protein.

1.3.12 SDS-PAGE analysis

An SDS-PAGE was performed to evaluate the structural integrity and molecular weight of HSA, cHSA, and cHSA-PNIPAAm. Two sample types were prepared: native and denatured. For the native samples, 2 µL of 1 mg/mL protein solution was mixed with 3 µL of loading buffer and 7 µL of Milli-Q water (no DTT), resulting in a total volume of 12 µL. For the denatured samples, 2 µL of 1 mg/mL protein solution was combined with 3 µL of loading buffer, 1.2 µL of DTT, and 5.8 µL of Milli-Q water, yielding a total volume of 12 µL. The denatured samples were heated at 95 °C for 30 min to ensure complete denaturation. Subsequently, each sample was loaded onto the SDS-PAGE gel. Following electrophoresis, the gel was stained with Coomassie Brilliant Blue to

visualize the protein bands.

1.3.13 Proteinosomes-based artificial cells (ACs) preparation

A 60 μL aliquot of a 5 mg/mL cHSA-PNIPAAm bioconjugate solution was first prepared in a 10 mM carbonate buffer (pH 8.5). Subsequently, 1.2 mg of PEG-bis (N-succinimidyl succinate) (Mw = 2000 Da) was introduced into the solution. For ACs encapsulated enzymes, add LOx (10 μg/μL, 2.5 μL) and PDC (5 μg/μL, 5 μL) into the solution, and the followed procedures were the same. This mixture was then combined with 1000 μL of 2-ethyl-1-hexanol, maintaining an aqueous-to-oil volume ratio of 0.06, and was vigorously agitated by pipetting 30 times. The resulting emulsion was incubated in the dark overnight. Next, a phase transfer procedure was followed to remove the oil phase. Initially, the transparent upper phase was discarded, and the remaining dispersion was extracted with 1 mL of 70% ethanol followed by centrifugation at 5000 g for 5 min. This extraction step was subsequently repeated with 40% ethanol (5000 g, 5 min), then with 20% ethanol (5000 g, 10 min), and finally with Milli-Q water (5000 g, 10 min). The final aqueous dispersion of proteinosomes-based ACs was collected and stored at 4 °C for further experiments.

1.3.14 SEM, AFM samples preparation

Scanning electron microscopy (SEM) samples were obtained through dropping 0.2 μg/μL ACs solution on the Au substrate and drying for 12 hours. Then, the images were obtained through Zeiss GeminiSEM 560. Atomic force microscopy (AFM, Bruker Dimension ICON) was used to physically characterize the morphology of the ACs. Samples were obtained through dropping 0.2 μg/μL ACs solution on the mica substrate and dried in the fume hood for 30 mins, 2 hours or 24 hours. The samples were scanned under tapping mode using cantilever Arrow-NCR with 285 kHz resonance frequency and a normal spring constant of 42 N·m-1. Gwyddion 2.63 SPM data visualization and analysis software was used to process the AFM images.

1.3.15 Stability of proteinosomes-based ACs in different environment

Samples were prepared by diluting ACs with ultrapure water, DMEM with 10 % FBS or different concentrations (1, 10, 100 μM) of H_2O_2 respectively to get 0.5 mg/mL ACs. DMEM and FBS were purchased from Gibco Life Technologies (Karlsruhe, Germany). H_2O_2 preparation: Using a commercial 30% w/w H_2O_2 stock (density 1.11 g/mL), pipette 10.2 μL of stock into 100 mL 50 mM PB (pH 7.4) to make a 1 mM stock solution. And then use a 1:10 dilution to obtain 100 μM solution, then dilute 100 μM to 10 μM and 10 to 1 μM. Add the samples solution into IBIDI 18-well glass bottom for further imaging. Confocal Laser Scanning Microscopy was performed on a STELLARIS 8 Leica DMi8 microscope (Leica Microsystems, S/N: 8300000313) with a 20x objective.

1.3.16 Confocal of LOx-Cy3 and PDC-Cy5 encapsulation

Labeling the enzymes: Adding 4.35 uL of Cy3-NHS (20 μg/μL in DMSO) into 2 μg/μL of LOx (1074 μL) in 1 mL 50 mM PB (pH 8.0). Take the PDC stock suspension, centrifuged it and removed the supernatant, dissolved the precipitation in 50 mM pH 8.0 PB to prepare 2 μg/μL of PDC solution. Adding 5.56 uL of Cy5-NHS (20 μg/μL in DMSO) into 2 μg/μL of PDC (1000 μL) in 1 mL 50 mM PB (pH 8.0). Gently mixing and incubating at room temperature for 4 hours with slow shaking (200 rpm). Unreacted dye was removed by ultrafiltration (30 kDa MWCO) with three washes using ultrapure water. Through testing the absorbance by Nanodrop, the information about dye and the formula: 1. Protein concentration = (A280-(Amax × CF)) × dilution factor / ε; 2. Moles dye per mole protein = Amax of the labeled protein × dilution factor / ε' × protein concentration. LOx-Cy3: $A_{280} = 0.254$, $A_{max} = 0.259$, $CF_{280} = 0.09$ was obtained from Lumiprobe website, dilution factor = 1, The extinction coefficient ($\varepsilon = 51{,}340\ M^{-1}$) was calculated from UniProt sequence Q44467 using ExPASy (ProtParam tool), molar extinction coefficient of the fluorescent dye ($\varepsilon' = 150{,}000\ M^{-1}$) was obtained from Lumiprobe website. About 0.4 moles dye modified on per mole protein. PDC-Cy5: $A_{280} = 0.264$, $A_{max} = 0.374$, $CF_{280} = 0.04$ was obtained from Lumiprobe website, dilution factor = 1, The extinction coefficient ($\varepsilon = 63{,}830\ M^{-1}$) was calculated from UniProt sequence P06169 using ExPASy (ProtParam tool), molar extinction coefficient of the fluorescent dye (ε' =

250,000 M^{-1}) was obtained from Lumiprobe website. About 0.4 moles dye modified on per mole protein. For the ACs preparation, following the procedures described in Section "1.3.13 Proteinosomes-based artificial cells (ACs) preparation". Dilute the P@LOx-Cy3, P@PDC-Cy5 and P/ LOx-Cy3, PDC-Cy5 into 0.5 μg/μL. For the P@LOx-Cy3 / P@PDC-Cy5 group, 30 μL of P@LOx-Cy3 and 30 μL of P@PDC-Cy5 were combined. Immediately after preparation (0 h) each sample was transferred to an IBIDI-Slide 18-well glass-bottom chamber and imaged by confocal microscopy. Samples were then incubated at room temperature and imaged again at 12 h and 24 h. Confocal Laser Scanning Microscopy was performed on a STELLARIS 8 Leica DMi8 microscope (Leica Microsystems, S/N: 8300000313) with a 20x objective.

1.3.17 Storage stability of free PDC/LOx and PDC/LOx encapsulated in proteinosome-based ACs

P@PDC/LOx were prepared and aged for the indicated times prior to purification (groups: 7 d, 1 d, 0 h). Free-enzyme controls were prepared by mixing LOx and PDC (freshly prepared) and storing the mixtures at 4 °C until the corresponding time points (7 days, 1 day, 0 hours). Immediately prior to the activity assay, proteinosomes were purified as described in Section "1.3.13 Proteinosomes-based artificial cells (ACs) preparation". After the final wash, P@PDC/LOx were prepared in 50 mM phosphate buffer (PB, pH 6.4) containing 40 mM lactate such that each 100 μL reaction contained 56.8 μg ACs, corresponding to 3.36 μg PDC and 1μg LOx. For the free-enzyme controls, reaction mixtures contained 1 μg LOx and 1 μg PDC in 100 μL 50 mM PB with 40 mM lactate. Reaction tubes were incubated at 37 °C for 2 hours. Acetaldehyde concentrations were then determined according to Section 1.3.4 using the colorimetric aldehyde assay kit (MAK139). Then, the acetaldehyde production was normalized to that of the freshly prepared sample (day 0) to quantify the retained activity over time.

1.3.18 Cytotoxicity of proteinosomes-based ACs on 4T1 and L929 cells

For cellular studies, commercially available 4T1 mammary carcinoma cells (ATCC, #CRL-2539) and L929 mouse fibroblast cells (ATCC, #CCL-1) were cultured at 37 °C and 5 % CO_2 in DMEM (4.5g/L D-glucose, L-glutamine; Gibco by ThermoFisher Scientific, Germany), supplemented with 10 % FBS (Sigma–Aldrich, Germany) and 1 % Penicillin/Streptomycin (Invitrogen, Germany). The cell viability of 4T1 and L929 cells incubated with four different groups (empty ACs, ACs encapsulated PDC, LOx or PDC and LOx) was determined using the CellTiter method. 4T1 cells and L929 cells were seeded in 96-well half area microplate with a density of 5,000 cells per well for overnight. After that, the medium was replaced with 50 µL of DMEM medium containing serial dilutions of empty ACs (from 1.1 µg to 10.3 µg per well) and enzyme-loaded ACs (PDC: 0.07 µg to 0.61 µg per well; LOx: 0.02 µg to 0.18 µg per well). Then, the cells were incubated (37 °C, 5% CO_2) for another 24 hours. Then, cytotoxicity of 2D monolayer cells were detected by CellTiter-Glo® Luminescent Cell Viability Assay (Promega, G7570).

1.3.19 Co-culture with 4T1 and L929 2D cells

4T1 cells or L929 cells were seeded in IBIDI 8-well glass-bottom plates at a density of 30,000 cells per well and allowed to adhere overnight. The following day, the culture medium was replaced with 100 µL of DMEM containing 46 µg of empty ACs and enzyme-loaded ACs (comprising 2.7 µg of PDC and 0.8 µg of LOx). The plates were then incubated under standard conditions (37 °C, 5% CO_2) and imaged after 10 mins using confocal laser scanning microscopy (CLSM). Thereafter, the plates were returned to the incubator for an additional 24 h before another round of imaging with CLSM.

1.3.20 Live/dead staining

4T1 cells were seeded in 96-well plates at a density of 10,000 cells per well and allowed to adhere overnight. The following day, the culture medium was replaced with 100 µL of DMEM containing 11.5 µg of ACs, which included empty ACs and enzyme-loaded formulations: P@PDC (0.68 µg of PDC), P@LOx (0.2 µg of LOx), and P@PDC/LOX (comprising 0.68 µg of PDC and 0.2 µg of LOx).

Subsequently, fluorescein diacetate (FDA) was diluted to 10 μg/mL and combined with propidium iodide (PI) at 2 μg/mL for live/dead cell staining. The medium was then replaced with this staining solution, and the cells were incubated under standard conditions for 30 min. After incubation, the cells were washed three times with PBS, and images were captured using a Keyence BZX800 microscope.

1.3.21 Development of spheroid–proteinosome co-culture systems

Two approaches were employed to establish spheroid–proteinosome co-culture models: the direct mixing method and the core–shell method, each implemented with or without Matrigel supplementation. For the direct mixing method, cells were first stained with CellTracker Green and subsequently mixed with Cy5-labeled ACs. A suspension of 10,000 cells was mixed with 11.5 μg of empty ACs or enzyme-loaded ACs (comprising 0.68 μg of PDC and 0.2 μg of LOx) per well was seeded in 96-well plates and centrifuged at 1200 rpm for 5 min. In the Matrigel group, after a 2-h incubation at 37 °C, 100 μL of complete medium containing 5% (v/v) Matrigel (cold, with a protein concentration of 11.1 mg/mL, resulting in a final concentration of 2.5%) was added to each well, followed by a second centrifugation at 1000 rpm for 5 min at 4 °C. The co-cultures were then maintained for one week. For the core–shell method, 10,000 cells per well were seeded and centrifuged at 1200 rpm for 5 min, and the spheroids were cultured for six days. On the subsequent day, the complete medium was replaced with 100 μL of medium containing Cy5-labeled ACs (11.5 μg of empty ACs or enzyme-loaded ACs contains 0.68 μg of PDC and 0.2 μg of LOx), and the plate was centrifuged at 1200 rpm for 5 min. For the Matrigel variant, complete medium containing 5% Matrigel (final concentration 2.5%) was then added to each well, followed by centrifugation at 1000 rpm for 5 min at 4 °C. These co-cultures were further maintained for one day prior to downstream analysis.

1.3.22 Live dead staining of spheroid–proteinosome co-culture systems

For this experiment, the core–shell method was employed with Matrigel supplementation: 10,000 cells per well were seeded and centrifuged at 1200 rpm for 5 min, and the spheroids were cultured for six days. On the subsequent day, the complete medium was replaced with 100 μL of medium

containing 46 μg of empty ACs or enzyme-loaded ACs contains 2.7 μg of PDC and 0.8 μg of LOx, and the plate was centrifuged at 1200 rpm for 5 min. Then, complete medium containing 5% Matrigel (final concentration 2.5%) was then added to each well, followed by centrifugation at 1000 rpm for 5 min at 4 °C. After that, the plate was put into incubator again for 24 hours incubation. After the 24 hours incubation, FDA was diluted to 10 μg/mL and combined with PI at 2 μg/mL. The culture medium was subsequently replaced with this staining solution, and the cells were incubated under standard conditions for 45 min. After incubation, the spheroids were washed three times with PBS, and images were captured using a Keyence BZX800 microscope.

1.3.23 Statistical analysis

All statistical analyses were performed using IBM SPSS Statistics. For comparisons between two groups, an unpaired two-tailed Student's t-test was used. For comparisons among more than two groups, one-way ANOVA was applied. Homogeneity of variance was assessed by Levene's test: if Levene's $p > 0.05$ (equal variances), LSD's post-hoc test was used for pairwise comparisons; if Levene's $p \leq 0.05$ (unequal variances), the Games-Howell post-hoc test was applied. In all tests, $p < 0.05$ was considered statistically significant. Data are presented as mean ± S.E.M. from at least three technical replicates ($n = 3$).

2. Supporting figures

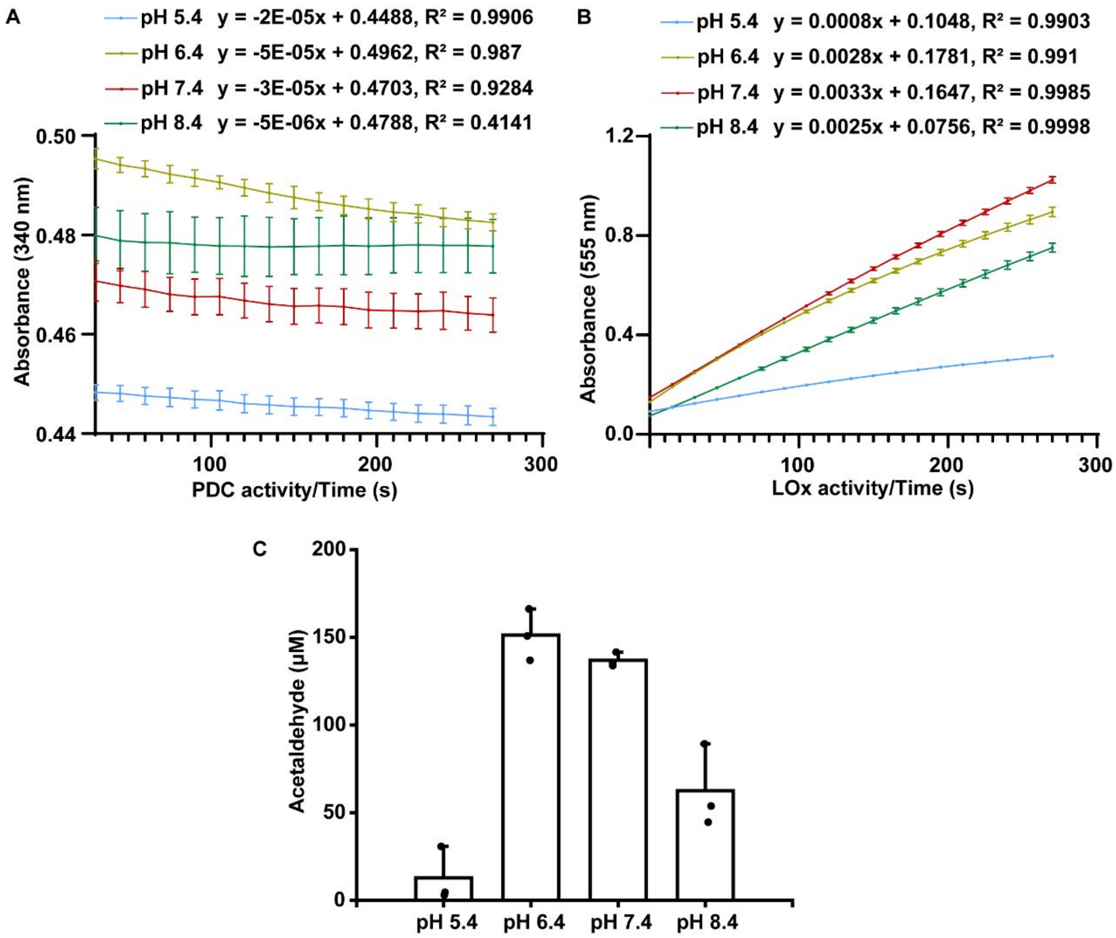

Figure S1. The catalytic activity of PDC **(A)** and LOx **(B)** in the buffers with pH values of 5.4, 6.4, 7.4, and 8.4. **C.** The concentrations of produced acetaldehyde resulted from PDC and LOx in lactate-contained buffers with pH values of 5.4, 6.4, 7.4, and 8.4.

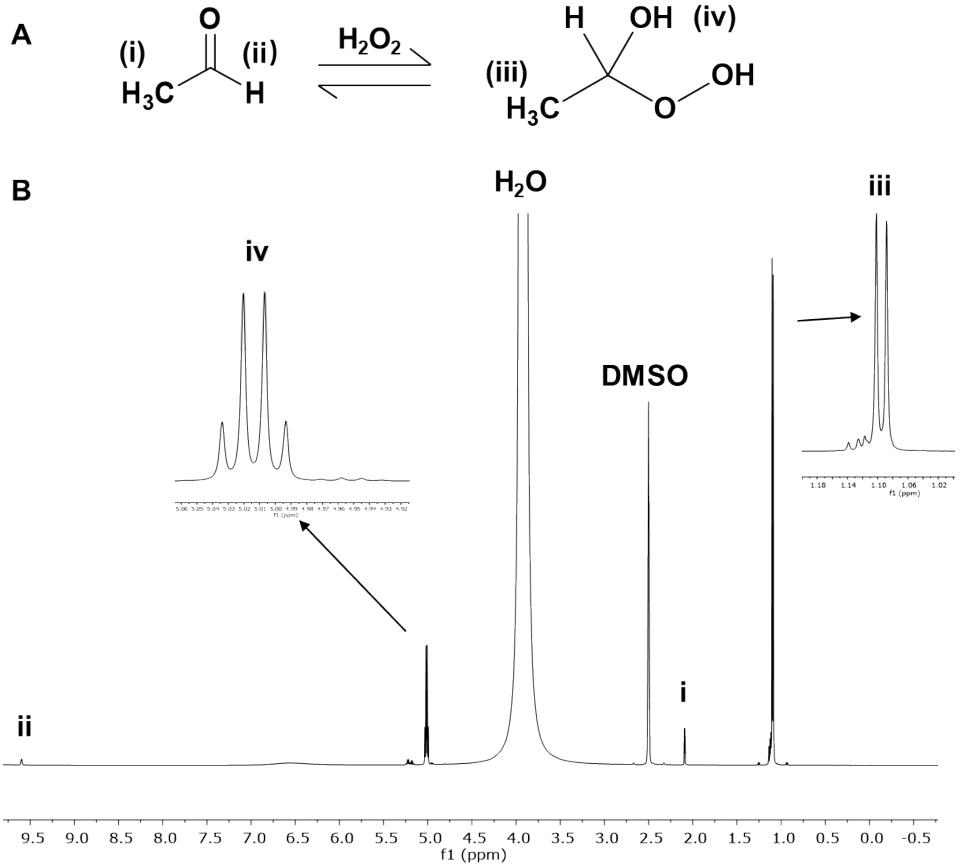

Figure S2. ^{1}H NMR spectra of acetaldehyde and H_2O_2 mixture in d_6-DMSO. The split pattern and the identity of each peak are shown in the figure (the numbers match those in the chemical structures).

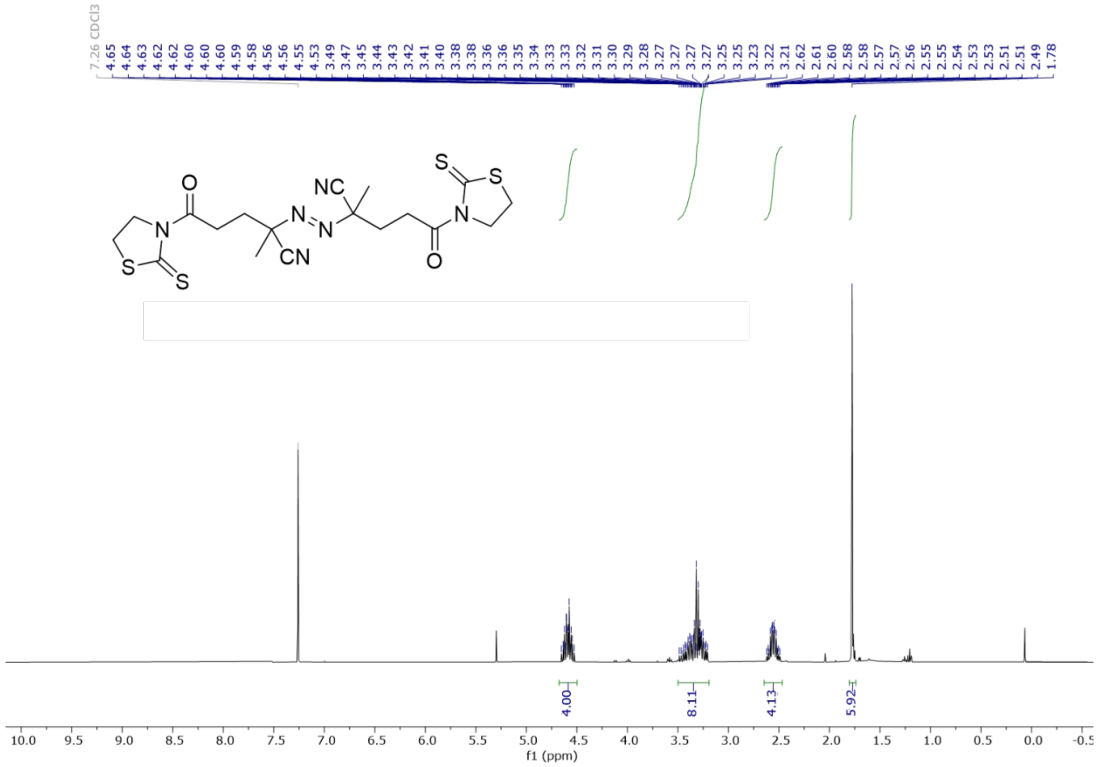

Figure S3. ^{1}H NMR spectrum of mercaptothiazoline activated 4,4'-azobis (4-cyanovaleric acid) (ACVA-ACPM) in $CDCl_3$.

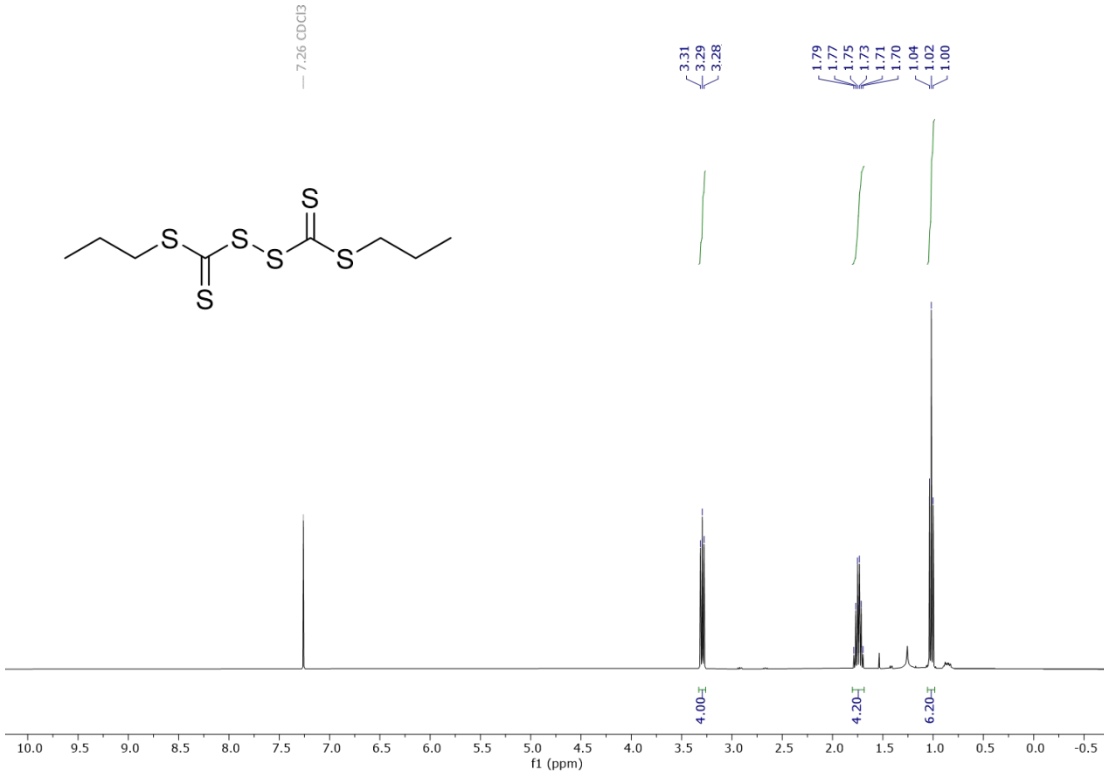

Figure S4. ^{1}H NMR spectrum of bis(propylsulfanylthiocarbonyl) disulfide in $CDCl_3$.

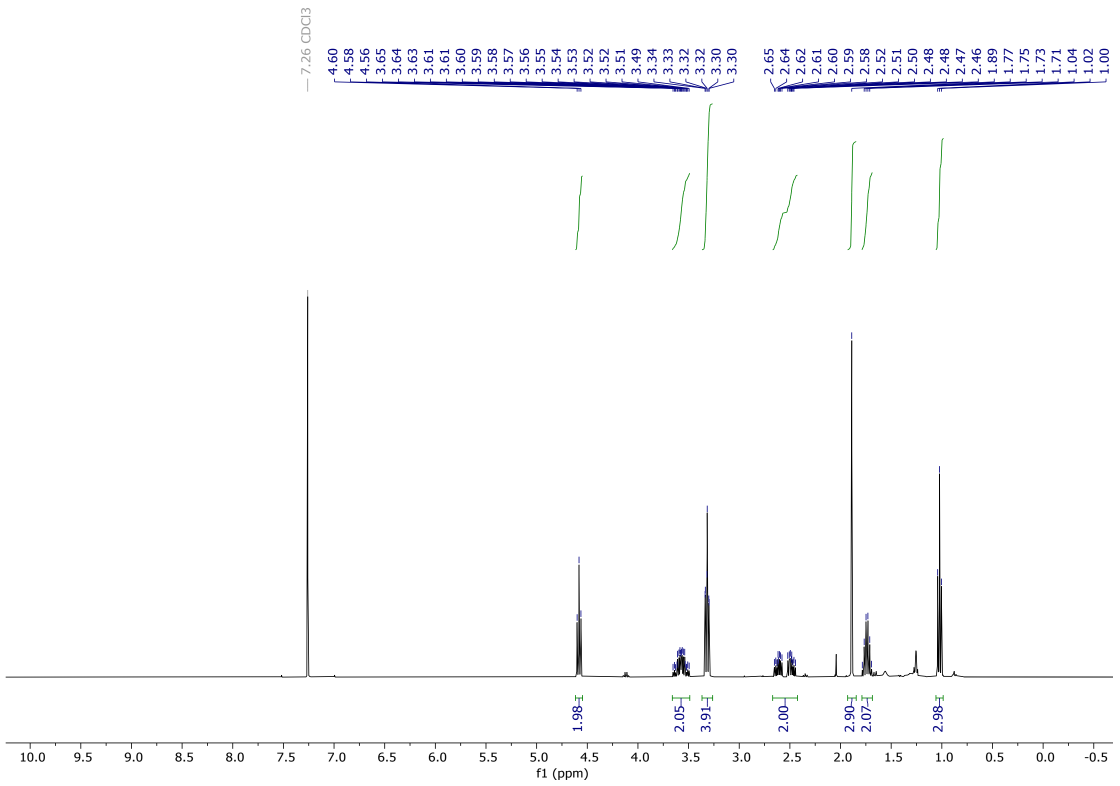

Figure S5. ^{1}H NMR spectrum of mercaptothiazoline-activated trithiol-RAFT agent 4-cyano-4-(propyllsulfanylthiocarbonyl) sulfanylpentanoic acid mercaptothiazoline amide in $CDCl_3$.

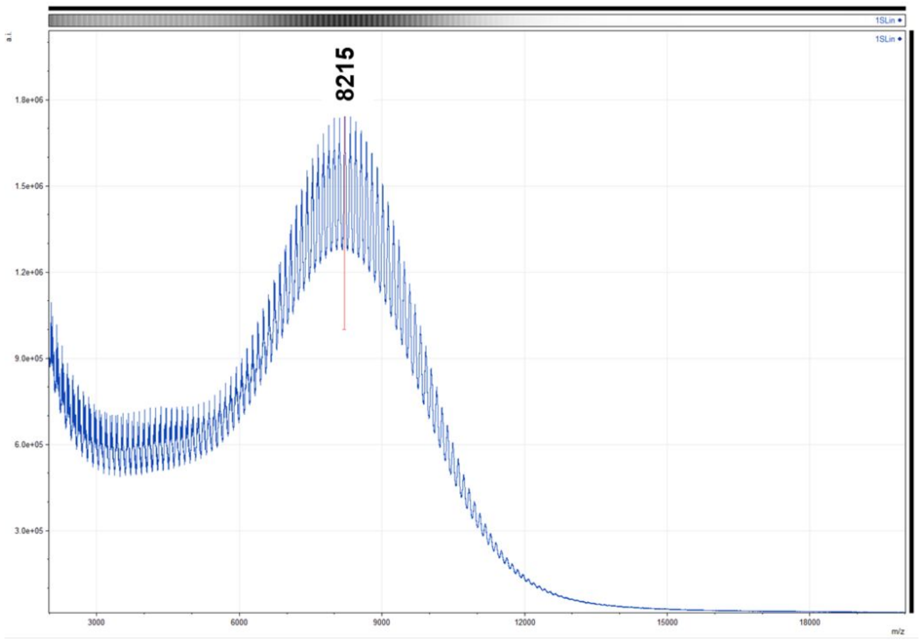

Figure S6. MALDI -Tof spectrum of PNIPAAm.

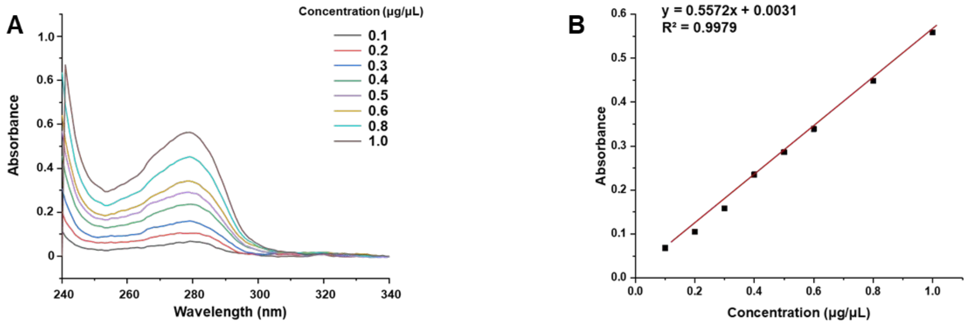

C

	Concentrations (μg/μL)	Absorbance (280nm)	PNIPAAm Content (cHSA/PNIPAAm)
Sample 1	0.6	0.289	1.41
Sample 2	0.4	0.191	1.53
Sample 3	0.24	0.112	1.82
Sample 4	0.12	0.060	1.29
Average			1.51±0.23

Figure S7. A. UV-vis spectra of native HSA at different concentrations from 0.1 to 1.0 μg/μL in an aqueous solution. **B.** Calibration curve for native HSA determined by plotting the UV-vis absorbance at 280 nm against concentration. Standard equation: y=0.5572x+0.0031, R2=0.9979. **C.** The number of PNIPAAm in cHSA/ PNIPAAm conjugates based on UV-vis spectroscopic analysis of four different sample concentrations.

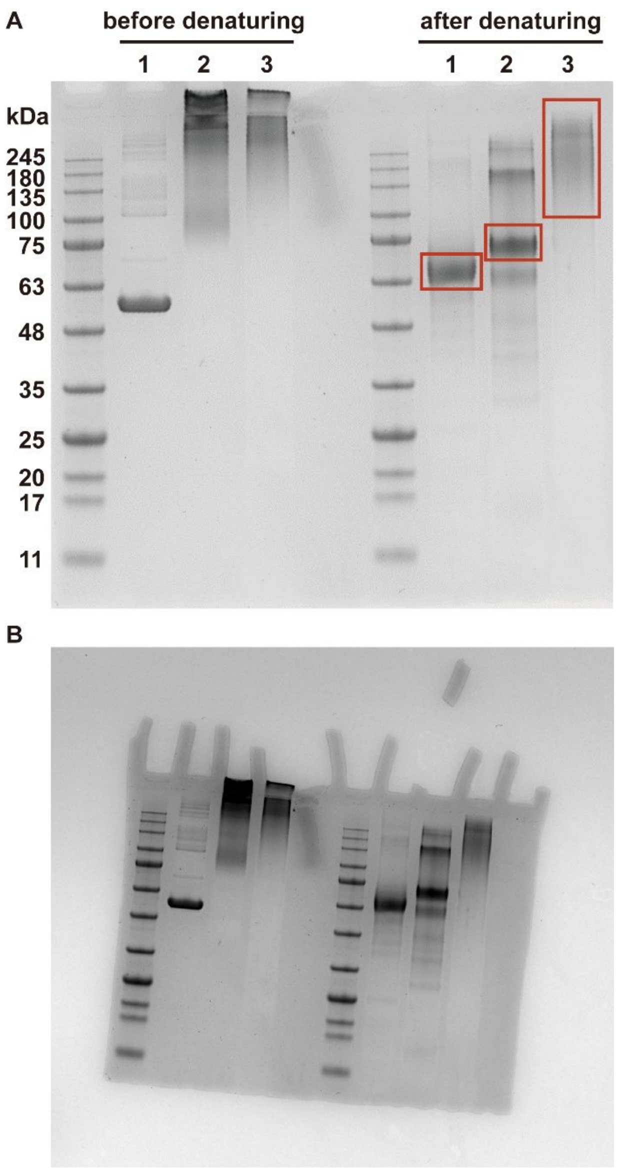

Figure S8. A. SDS-PAGE validation of covalent cHSA-PNIPAAm conjugation: Lane 1 (native HSA, Mw=66,500 g/mol), Lane 2 (cHSA, Mw=72,800 g/mol), Lane 3 (cHSA-PNIPAAm, Mw=85,122 g/mol). (The red box highlights the migration position of each protein, loading amount: 2 µg sample/well). **B.** Uncropped and unadjusted original SDS-PAGE image, shown without modifications to brightness or contrast.

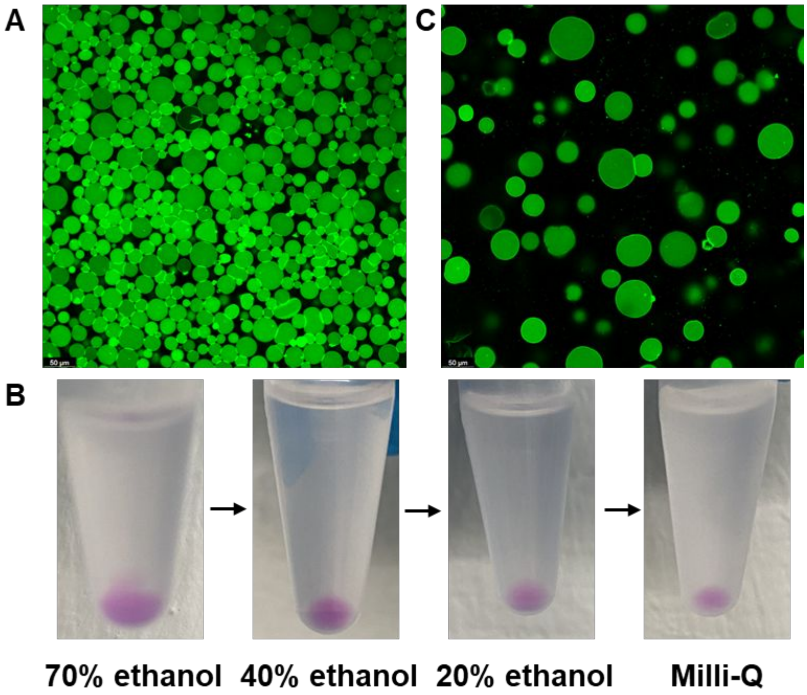

Figure S9. A. Proteinosomes-based ACs in oil phase, scale bar = 50 μm. **B.** ACs were purified in 70%, 40%, and 20% ethanol, as well as in Milli-Q water. Precipitation was observed following centrifugation in these environments. To enhance the visibility of the precipitation, HSA-Cy5 was encapsulated within the ACs. **C.** ACs in water phase, scale bar = 50 μm.

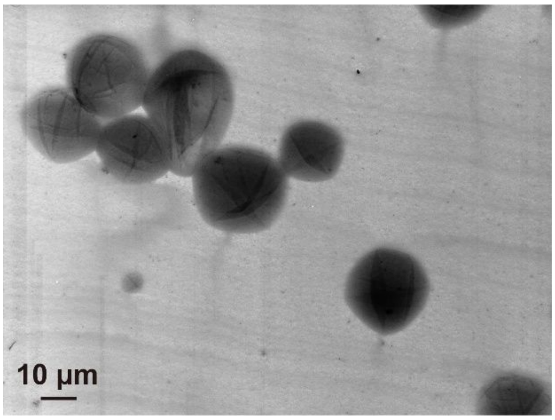

Figure S10. SEM images for showing the structure of ACs. Scale bar = 10 μm. (0.2 μg/μL ACs solution on the Au substrate, dried for 12 h).

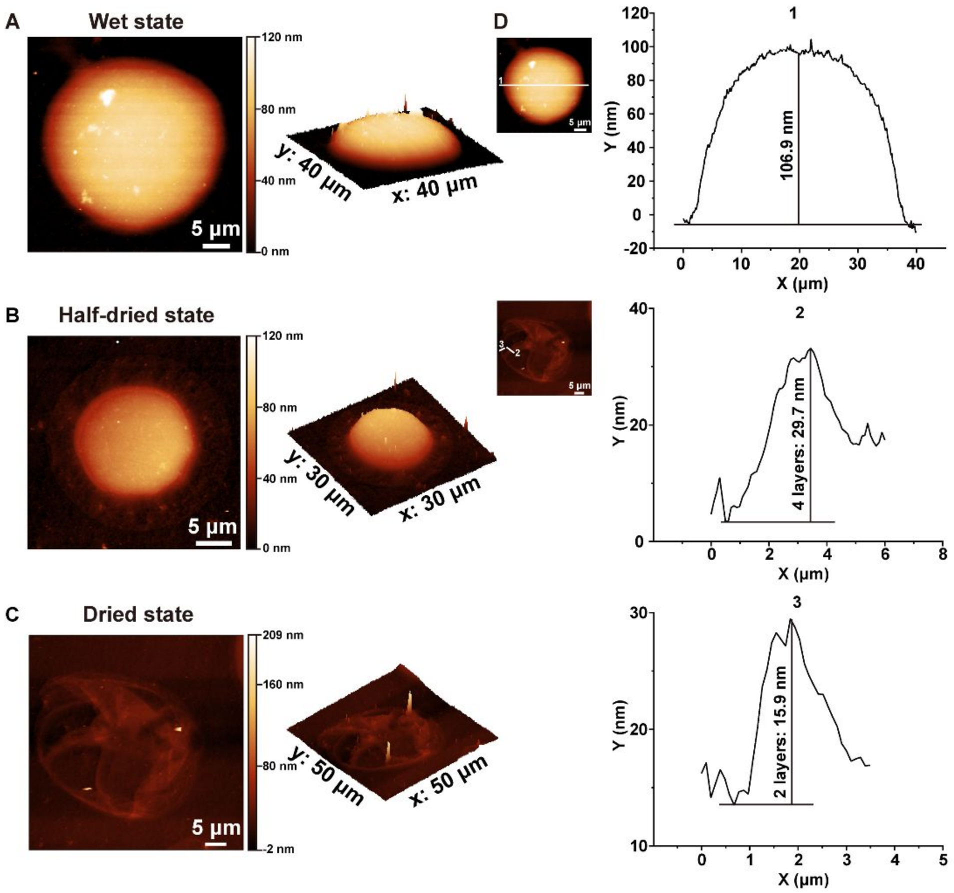

Figure S11. 2D and 3D AFM topological maps: **A.** hydrated state, **B.** half-dried state, **C.** dried state. Scale bar = 5 µm. (0.2 µg/µL ACs solution on the mica substrate and dried for A. 30 mins, B. 2 h and C. 24 h). **D.** AFM images after analysis. Corresponding height profile along the white lines (1, 2, 3) that traverses liquid-contained height, double and quadruple membrane thicknesses after dried.

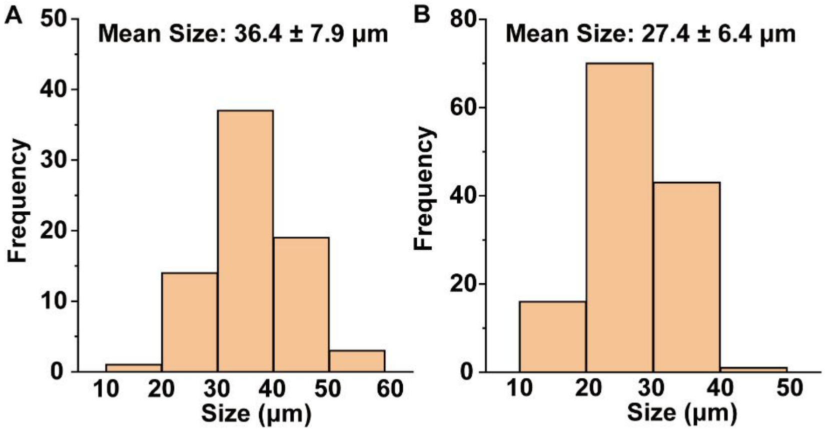

Figure S12. Plot showing mean size of ACs dispersed in DMEM with 10 % FBS at 37 ℃ on the day 0 **(A)** and day 7 **(B)**. Sample sizes: n = 74 for day 0 **(A)** and n = 130 for day 7 **(B)**, derived from three randomly selected CLSM fields under identical culture conditions.

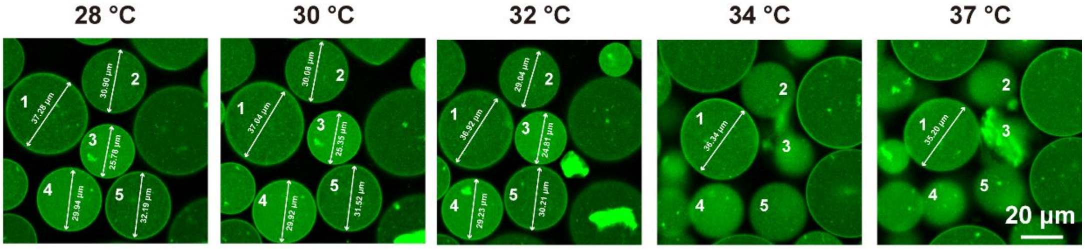

Figure S13. Confocal microscopy images of ACs recorded at temperatures ranging from 28°C to 37°C, illustrating temperature-induced variations in size. The diameter of proteinosome 1 decreased slightly by 5.6 %, from 37.3 μm at 28 ℃ to 35.2 μm at 37 ℃; ACs 2-5 exhibited an average diameter decrease of 4.7 %, from 29.7 μm at 28°C to 28.3 μm at 32 ℃. Scale bar = 20 μm.

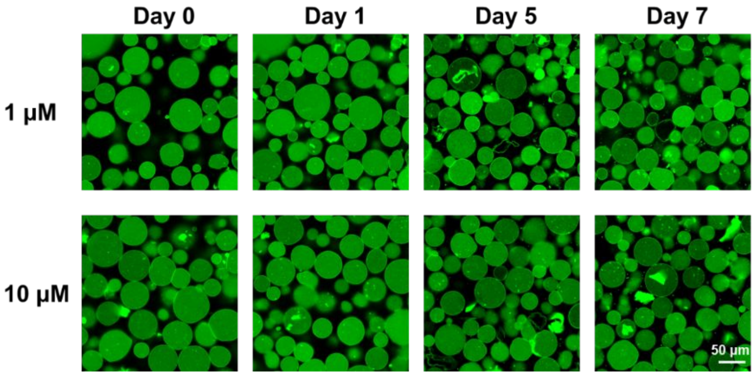

Figure S14. Confocal microscopy images of ACs under 1 or 10 µM H_2O_2 conditions up to 7 days. Scale bar = 20 µm.

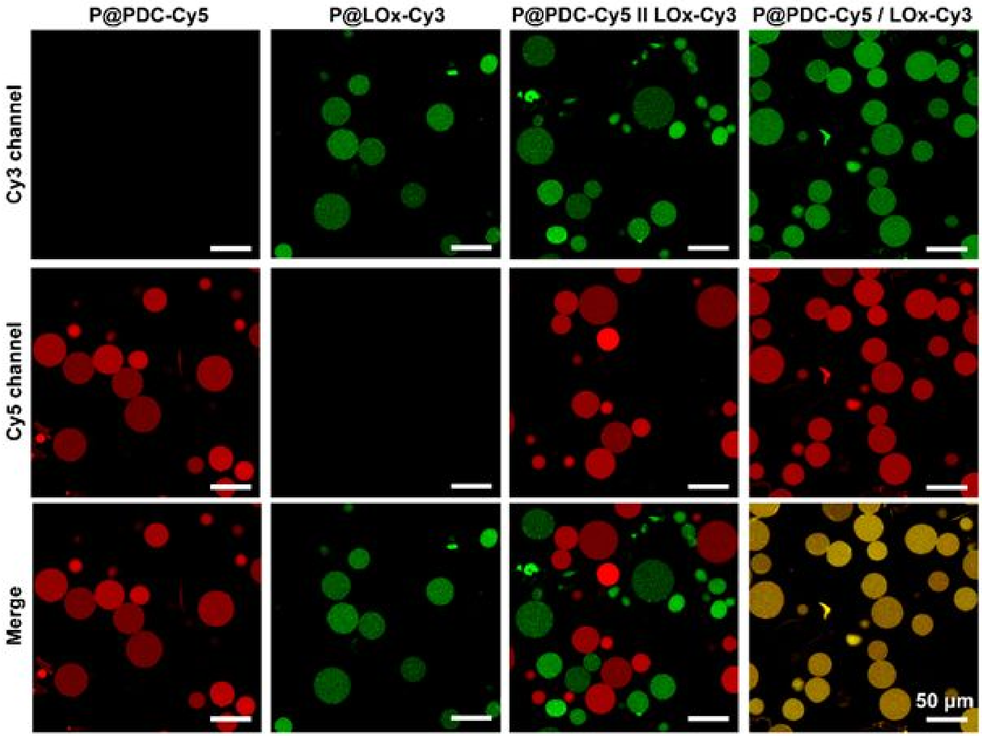

Figure S15. Confocal microscopy images of four different AC designs (0.5 µg/µL ACs suspended in Milli-Q). Cy5-labeled PDC (red) and Cy3-labeled LOx (green) exhibit overlapping signals when co-encapsulated (P@PDC/LOx ACs (merged)). In contrast, mixed AC populations (P@PDC‖LOx) containing the individual enzymes retain their red and green signals (group P@PDC‖LOx). All groups were images directly after being prepared. Scale bar = 50 µm

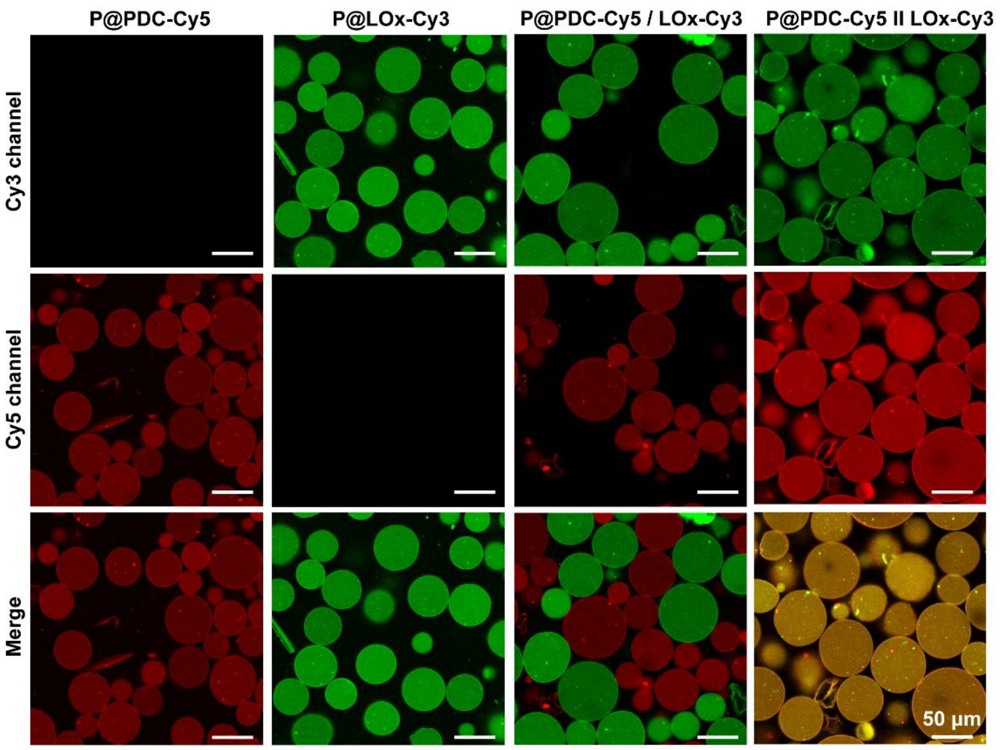

Figure S16. Confocal microscopy images of four different AC designs (0.5 µg/µL ACs suspended in Milli-Q). Cy5-labeled PDC (red) and Cy3-labeled LOx (green) exhibit overlapping signals when co-encapsulated (P@PDC/LOx ACs (merged)). In contrast, mixed AC populations (P@PDC‖LOx) containing the individual enzymes retain their red and green signals (group P@PDC‖LOx). All groups were images directly after 12 hours of incubation. Scale bar = 50 µm

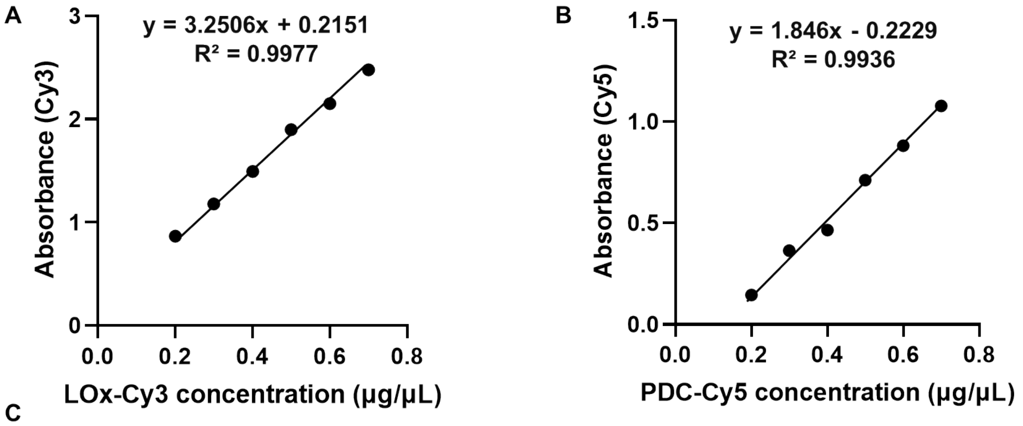

C

	Proteinosome/LOx-Cy3 Encapsulation efficiency (%)	Proteinosome/PDC-Cy5 Encapsulation efficiency (%)	Proteinosome/LOx-Cy3 and PDC-Cy5 Encapsulation efficiency (%)	
	LOx-Cy3	PDC-Cy5	LOx-Cy3	PDC-Cy5
Sample 1	26.3	77.4	20.6	66.5
Sample 2	24.6	71.4	21.4	71.4
Sample 3	27.0	73.3	21.5	75.0
Average	26.0 ± 1.2	74.0 ± 3.1	21.1 ± 0.5	71 ± 4.3

Figure S17. A. Calibration curve for LOx-Cy3 determined by plotting the UV-vis absorbance at 550 nm against concentration. Standard equation: y=3.2506x+0.2151, R^2=0.9977. **B.** Calibration curve for PDC-Cy5 determined by plotting the UV-vis absorbance at 650 nm against concentration. Standard equation: y=1.846x-0.2229, R^2=0.9936. **C.** The enzyme encapsulation based on the absorbance intensity of LOx-Cy3 and PDC-Cy5.

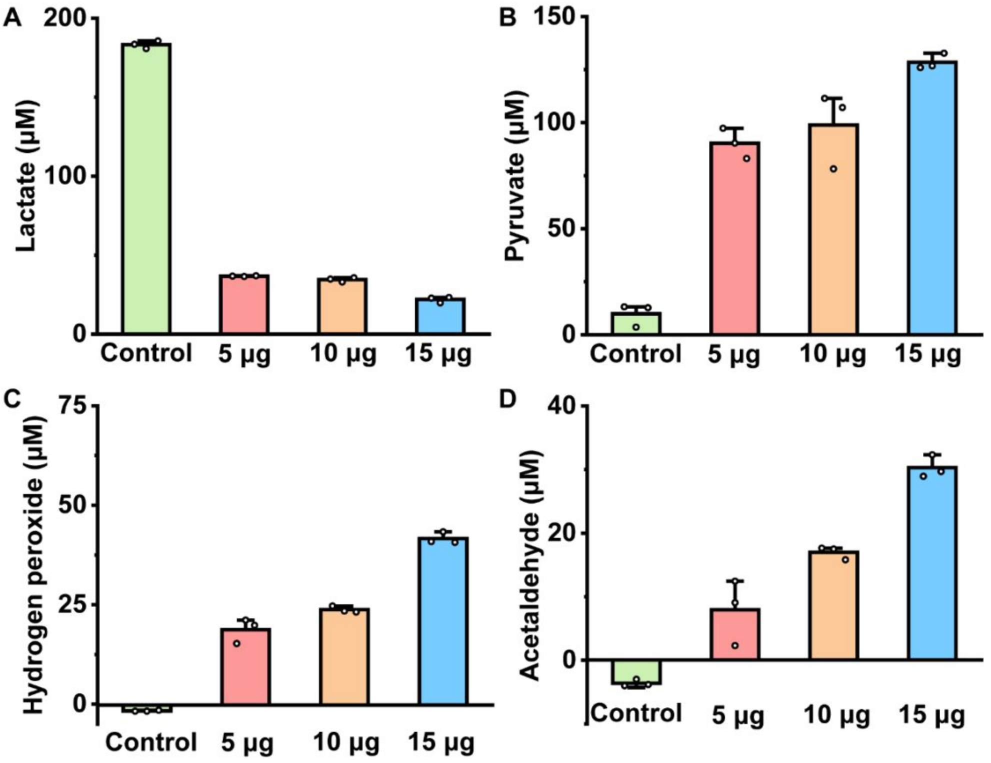

Figure S18. A. Lactate concentrations following treatment with 5, 10, 15 μg of P@PDC/LOX. Pyruvate **(B)**, H_2O_2 **(C)**, acetaldehyde **(D)** concentrations following treatment with 5, 10, 15 μg P@PDC/LOX in 50 μL PB (50 mM, pH 6.4, final concentration: 100, 200, 300 μg/mL P@PDC/LOX). Values are plotted as mean ± S.E.M (n = 3).

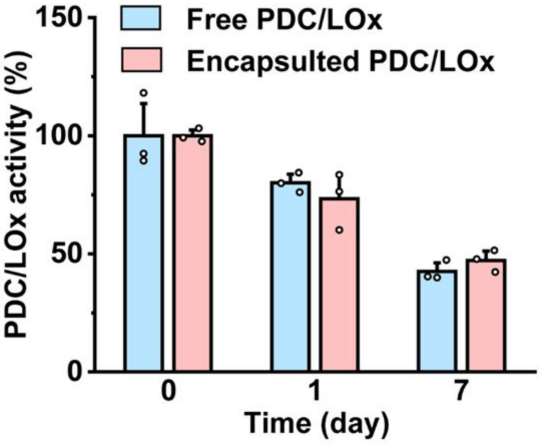

Figure S19. Storage stability of P@PDC/LOx and free PDC/LOx monitored *via* acetaldehyde production. The samples were stored at 4 °C and acetaldehyde was quantified at 0, 1 and 7 days. Acetaldehyde levels were normalized to the freshly prepared (0 h) sample. Values are plotted as mean ± S.E.M (n = 3).

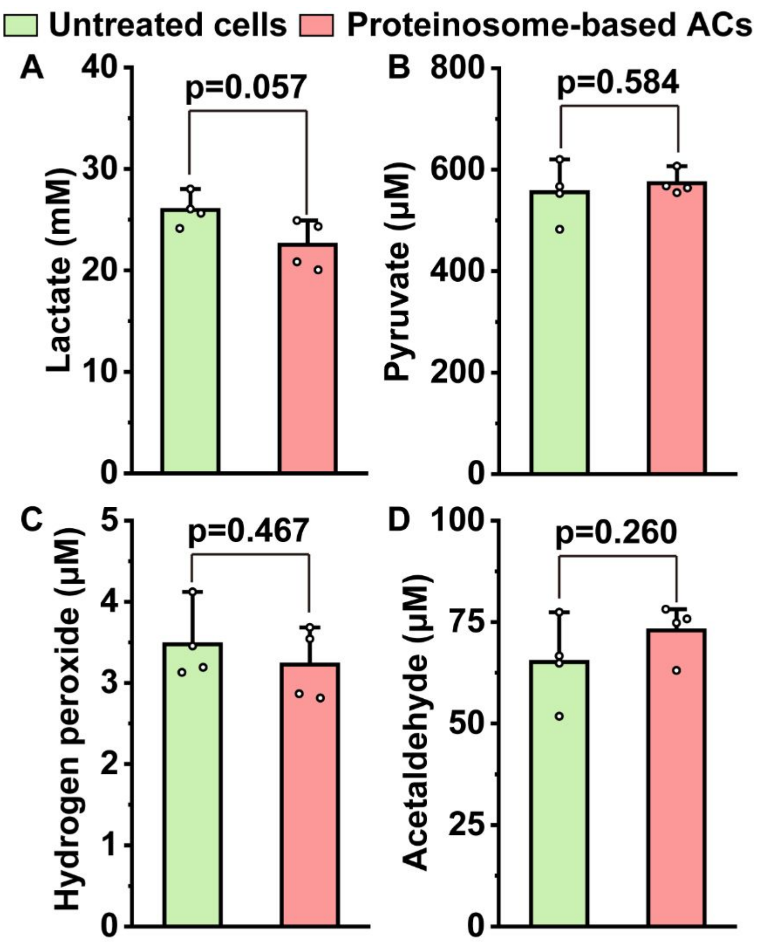

Figure S20. Associated indicator parameters: lactate **(A)**, pyruvate **(B)**, H_2O_2 **(C)**, acetaldehyde **(D)** concentrations of untreated cells and empty proteinosomes-treated cells. Values are plotted as mean ± S.E.M (n = 4). These data were analyzed by an independent t-test. $p > 0.05$ indicates no significant difference.

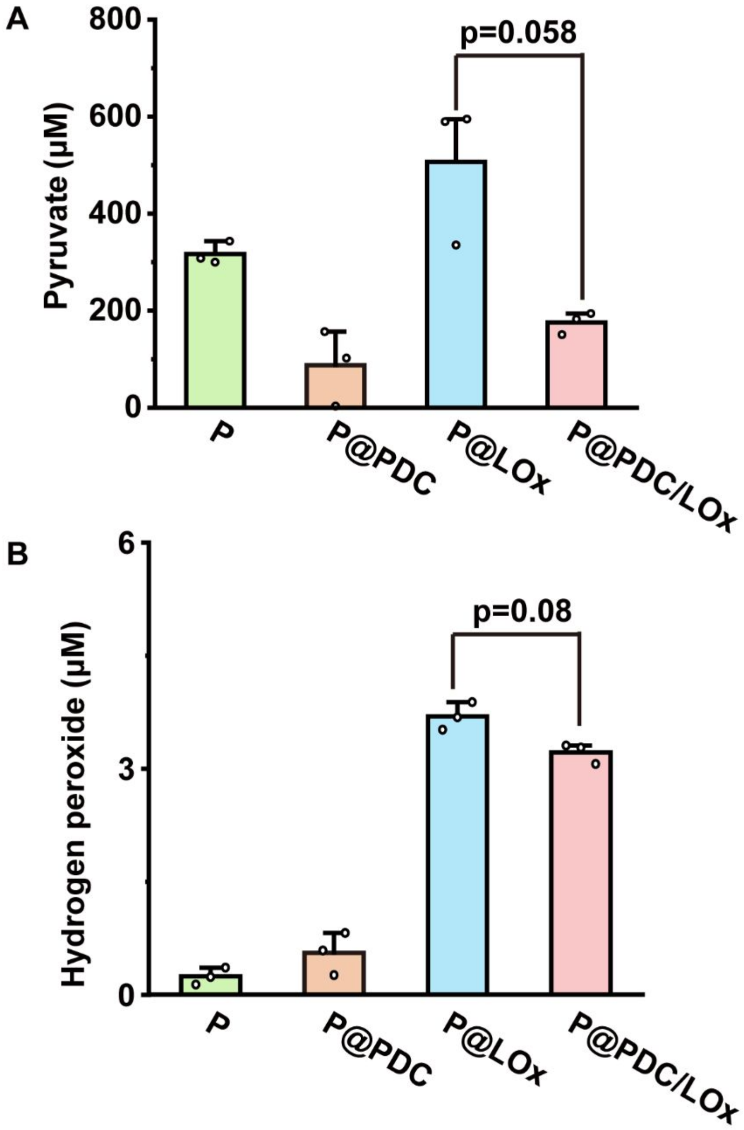

Figure S21. Pyruvate **(A)** and H_2O_2 **(B)** concentrations on 2D cell level after being treated with empty ACs, P@PDC, P@LOx and P@PDC/LOX respectively. Cells plated in 24-well plates were treated with: 1. empty ACs 46 μg, 2. P@PDC (44 μg ACs containing 2.7 μg PDC), 3. P@LOx (37 μg ACs containing 0.8 μg LOx), 4. P@PDC/LOX (46 μg ACs containing 2.7 μg PDC and 0.8 μg LOx). Values are plotted as mean ± S.E.M (n = 3). These data were analyzed by an independent t-test.

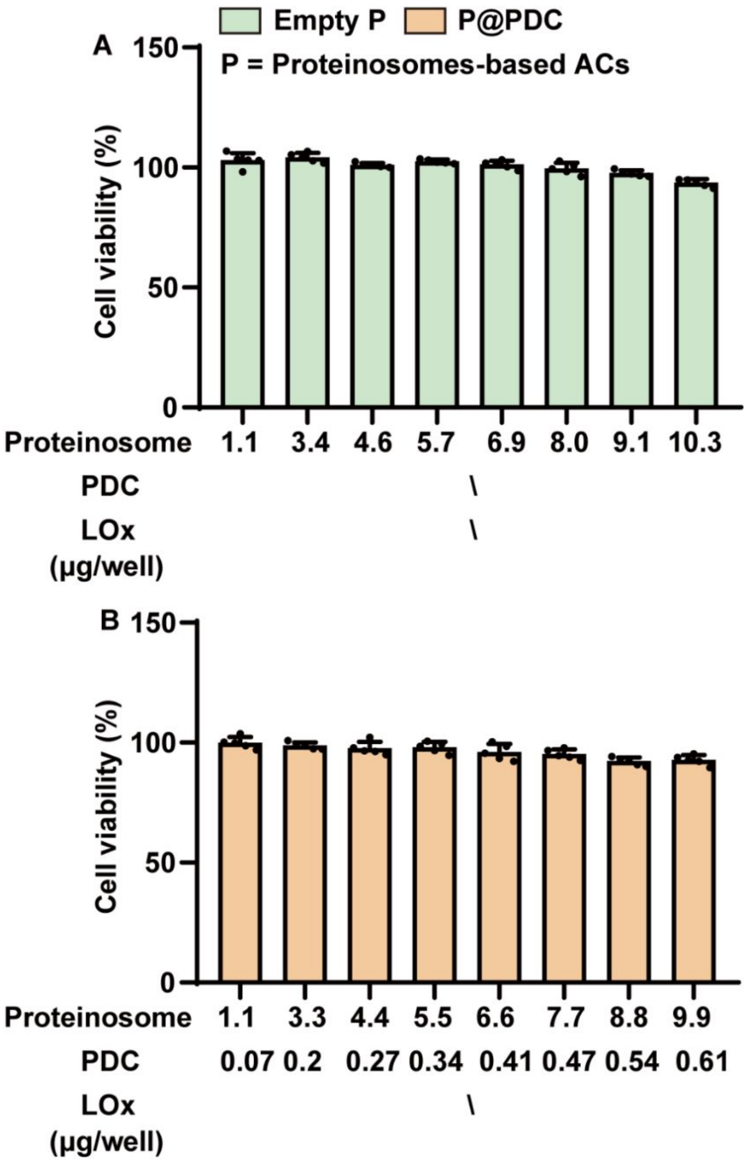

Figure S22. Cytotoxicity of 4T1 cell treated with different concentrations of empty ACs **(A)** and P@PDC **(B)**. Values are plotted as mean ± S.E.M (n = 5).

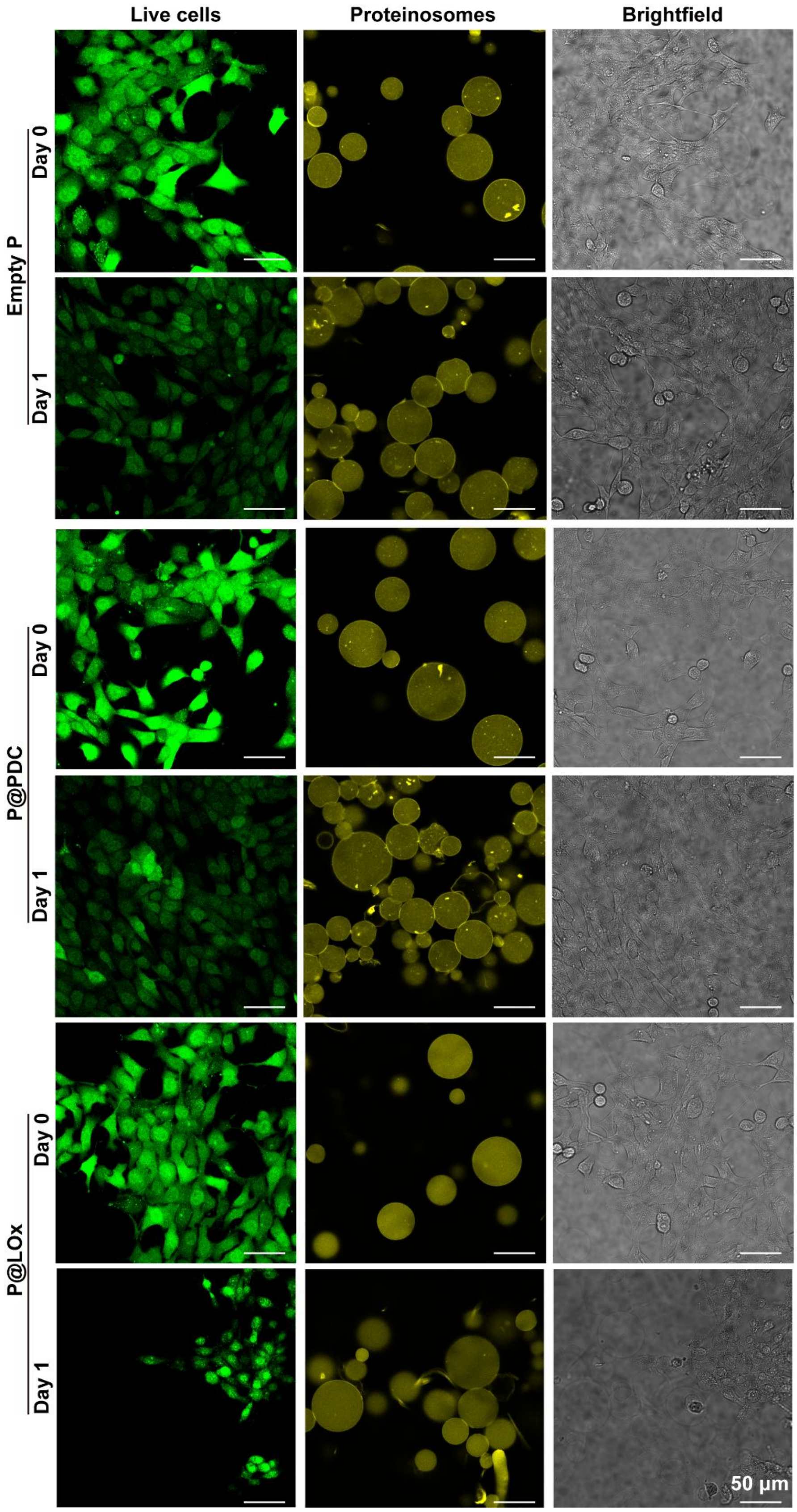

Figure S23. Confocal images of 4T1 cells incubated with empty P, P@PDC or P@LOx for 0 day and 1 day. Green channel: Cell tracker-green stained cells. Yellow channel: ACs. Scale bar = 50

μm.

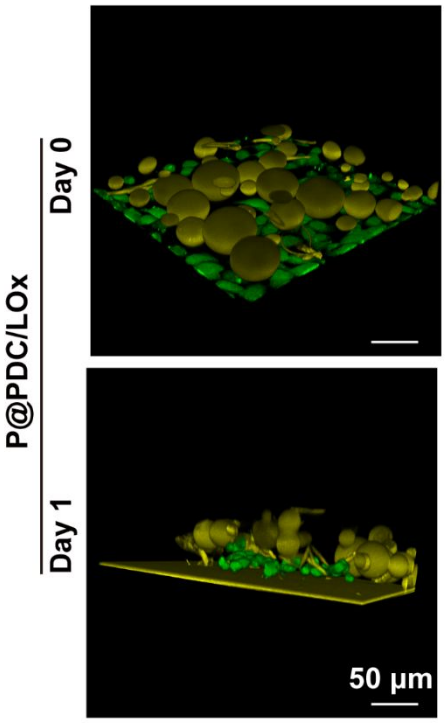

Figure S24. 3D images of P@PDC/LOX co-cultured with 4T1 cells for 0 day and 1 day. Green channel: Cell tracker-green stained cells. Yellow channel: ACs. Scale bar = 50 μm.

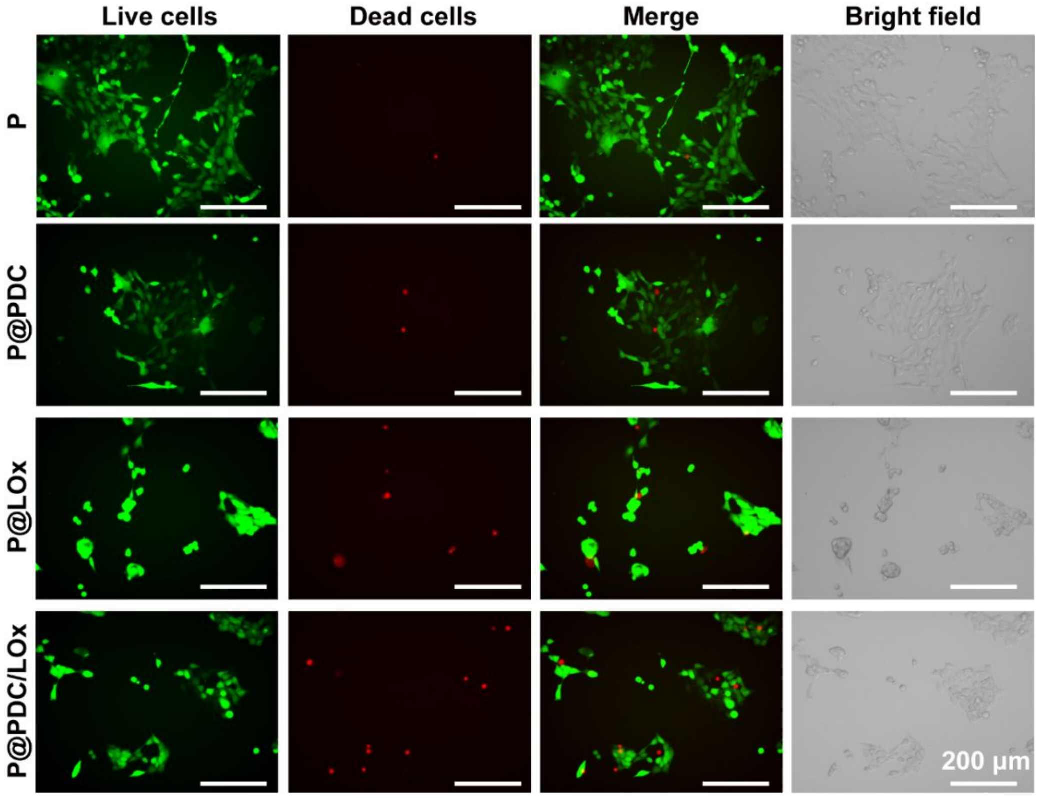

Figure S25. Live/Dead staining after four groups incubated with cells after 1 day. Scale bar = 200 μm.

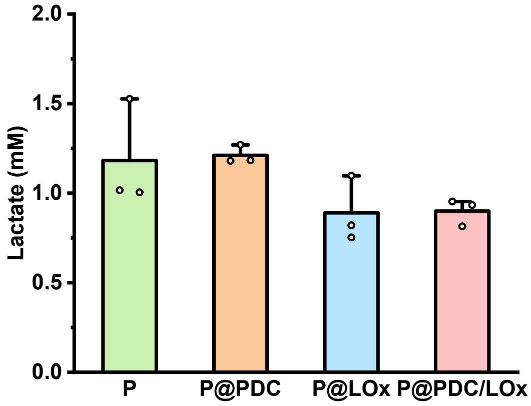

Figure S26. Extracellular lactate concentrations of L929 cells treated with empty ACs (P), P@PDC, P@LOx, or P@PDC/LOx. Values are plotted as mean ± S.E.M (n = 3).

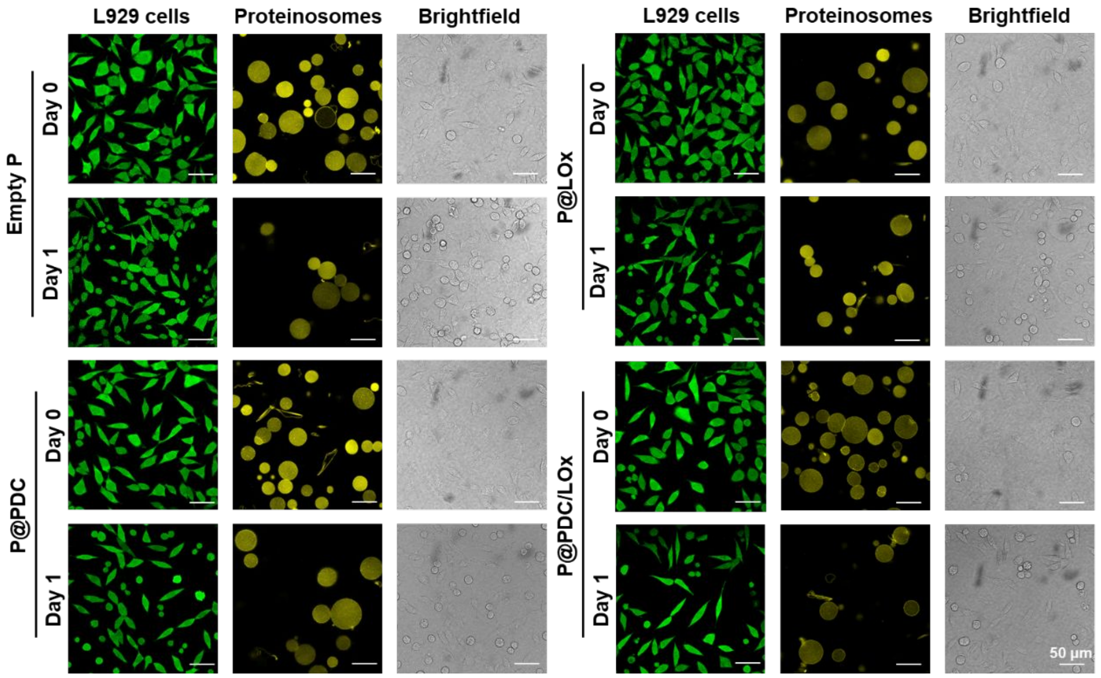

Figure S27. Confocal images of L929 cells incubated with empty P, P@PDC, P@LOx or P@PDC/LOx for 0 day and 1 day. Green channel: Cell tracker-green stained cells. Yellow channel: ACs. Scale bar = 50 μm.

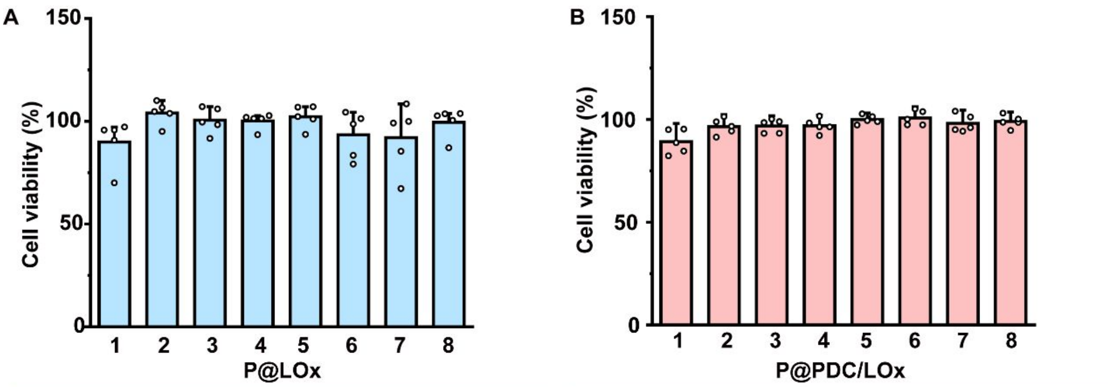

Group	P (μg)	PDC (μg)	LOx (μg)	CV (%)
1	0.9		0.02	89.9
2	2.8		0.06	104.1
3	3.7		0.08	100.5
4	4.6	\	0.1	100.2
5	5.5		0.12	102.2
6	6.5		0.14	93.4
7	7.4		0.16	92.0
8	8.3		0.18	99.5

Group	P (μg)	PDC (μg)	LOx (μg)	CV (%)
1	1.1	0.07	0.02	89.2
2	3.4	0.2	0.06	96.5
3	4.6	0.27	0.08	96.8
4	5.7	0.34	0.1	97.0
5	6.9	0.41	0.12	99.9
6	8.0	0.47	0.14	100.7
7	9.1	0.54	0.16	98.1
8	10.3	0.61	0.18	99.0

Figure S28. Cytotoxicity of L929 cell treated with different concentrations of P@LOx (0.9 μg with 0.02 μg LOx; incubation time, 24 h) and P@PDC/LOx (1.1 μg with 0.07 μg PDC and 0.02 μg LOx; incubation time, 24 h). Values are plotted as mean ± S.E.M (n = 5).

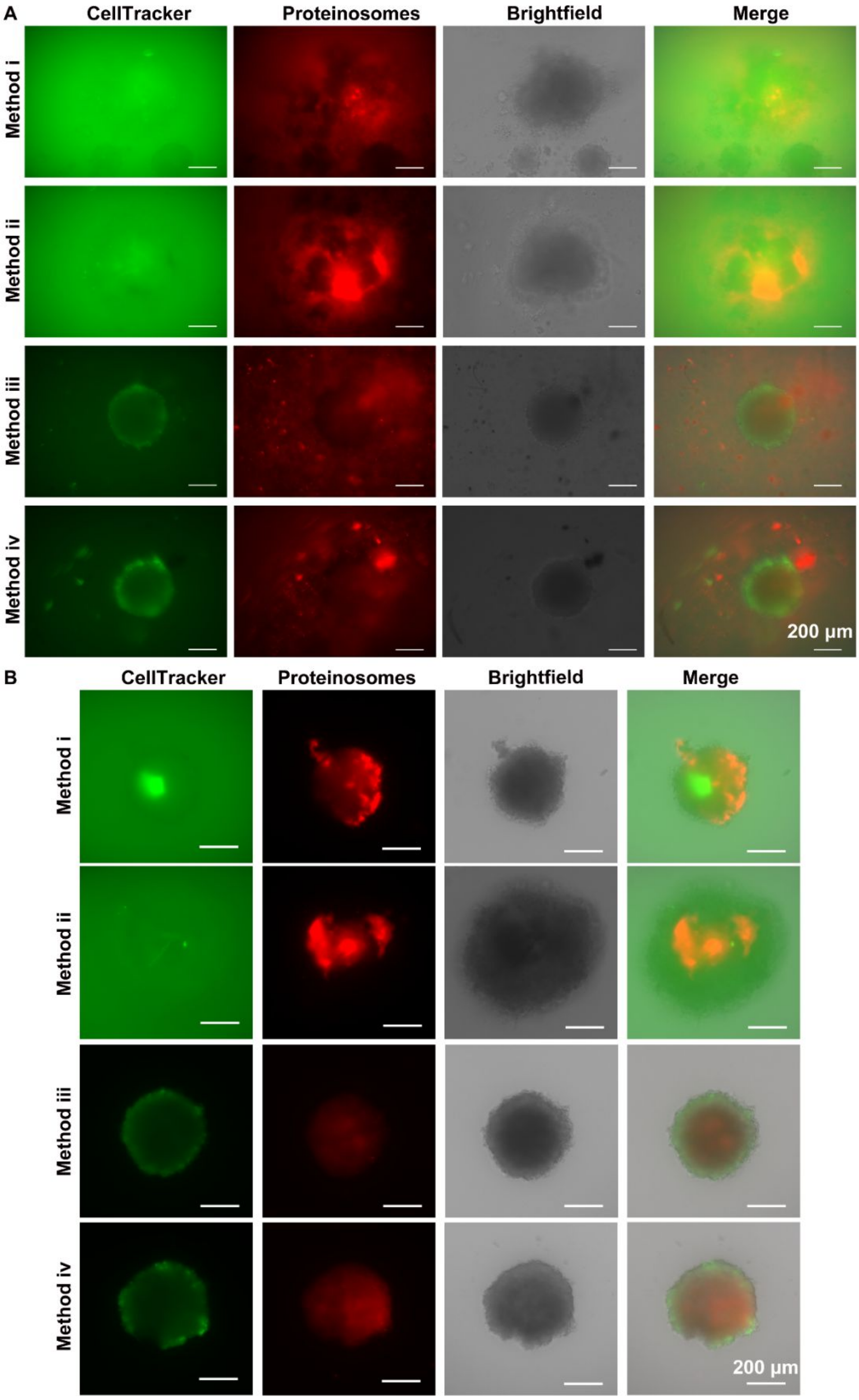

Figure S29. Three-dimensional model of proteinosome-based artificial cells co-culture with cancer cells before washing **(A)** and after washing **(B)** through four methods: **i)** Direct mixing without Matrigel. **ii)** Direct mixing with Matrigel. **iii)** Core-shell without Matrigel. **iv)** Core-shell with Matrigel. Scale bar = 200 μm

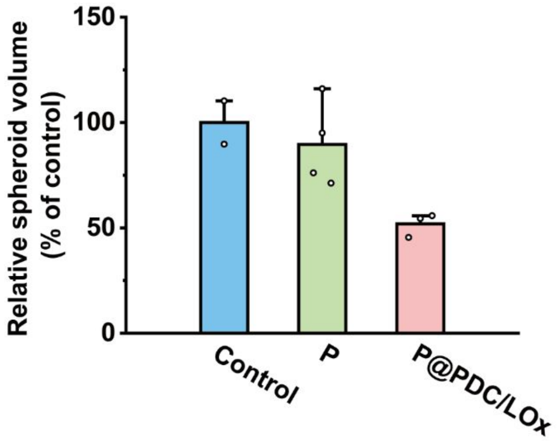

Figure S30. Analysis of spheroid volumes following treatment in different groups using AnaSP software. Relative spheroid volume was calculated as (treated group volume / control group volume) × 100%. The Empty ACs group (P) exhibited 90 ± 20 % relative volume, whereas the P@PDC/LOx group showed 52 ± 6 %. Values are plotted as mean ± S.E.M (n = 2 for control, n= 4 for P, n=3 for P@PDC/LOx).

3. Reference

[1] X. Huang, M. Li, D.C. Green, D.S. Williams, A.J. Patil, S. Mann, Interfacial assembly of protein–polymer nano-conjugates into stimulus-responsive biomimetic protocells, *Nat. Commun.* **2013**, *4*, 2239.

[2] N. Martin, J.-P. Douliez, Y. Qiao, R. Booth, M. Li, S. Mann, Antagonistic chemical coupling in self-reconfigurable host–guest protocells, *Nat. Commun.* **2018**, *9*, 3652.
